# Food resources affect territoriality of invasive wild pig sounders with implications for control

**DOI:** 10.1038/s41598-021-97798-z

**Published:** 2021-09-22

**Authors:** John C. Kilgo, James E. Garabedian, Mark Vukovich, Peter E. Schlichting, Michael E. Byrne, James C. Beasley

**Affiliations:** 1 USDA Forest Service, Southern Research Station, P.O. Box 700, New Ellenton, SC 29809 USA; 2 USDA Forest Service, Shawnee National Forest, 602 North First St., Vienna, IL 62995 USA; 3 Savannah River Ecology Laboratory, Warnell School of Forestry and Natural Resources, University of Georgia, P.O. Drawer E, Aiken, SC 29802 USA; 4 Illinois Department of Natural Resources, 1 Natural Resources Way, Springfield, IL 62702 USA; 5 School of Natural Resources, University of Missouri, Columbia, MO 65211 USA

**Keywords:** Biological techniques, Ecology, Zoology, Ecology, Natural hazards

## Abstract

Interest in control methods for invasive wild pigs (*Sus scrofa*) has increased due to their range expansion, population growth, and an improved understanding of their destructive ecological and economic effects. Recent technological advances in traps for control of pig populations facilitate capture of entire social groups (sounders), but the efficacy of “whole-sounder” trapping strategies is heavily dependent on the degree of territoriality among sounders, a topic little research has explored. We assessed territoriality in wild pig sounders on the Savannah River Site, South Carolina, USA, and examined whether availability of food resources provided by a municipal-waste landfill affected among-sounder territoriality. We estimated utilization distribution overlap and dynamic interactions among 18 neighboring sounders around a landfill. We found that although neighboring sounders overlapped in space, intensity of use in shared areas was uniformly low, indicating territorial behavior. Neighbors tended to share slightly more space when closer to the landfill waste cells, indicating availability of a super-abundant resource somewhat weakens the degree of territoriality among sounders. Nevertheless, we conclude that sounders behaved in a generally territorial manner, and we discuss implications for whole-sounder trapping programs, particularly near concentrated resources such as landfills and crop fields.

With the rapid increase in their distribution and abundance worldwide, wild pigs (*Sus scrofa*) have been recognized by the World Conservation Union (IUCN) as ranking among the 100 worst non-native invasive species in the world^1^, as well as one of the 10 most important invasive species and one of the two most important invasive terrestrial vertebrates in North America^2^. Even in Eurasia, where the species is native, wild boar distribution is expanding and population sizes are increasing, with impacts similar to those observed in the introduced range^3^. Such impacts include habitat destruction via rooting, depredation of tree seedlings, crops, and livestock, degradation of water quality, spread of disease, property damage, competition with and predation on native wildlife, and vehicle collisions^4^. Annual economic impact to agriculture alone in the U.S. is estimated at $1.5 billion^5^, a figure that may underestimate the damage considering the growth in wild pig populations since its publication.

Interest in control methods for wild pigs has increased concurrent with their rapidly expanding populations. Live trapping and removal is among the most widely used methods, but conventional traps frequently capture only a portion of a sounder (matrilineal social groups of one or more females and one or more generations of their offspring). Thus, such traps not only render control incomplete by leaving some pigs to continue reproducing, but also by increasing the likelihood those pigs not captured will become trap-shy and difficult to capture in future efforts^6^. Recently, the concept of “whole-sounder” trapping has garnered considerable attention as a potentially more effective trapping strategy for population control compared to conventional traps^6^. Whole-sounder trapping leverages the latest advances in trap design and remote camera technologies to facilitate capture of entire sounders at once. With precise information about sounder size and composition obtained prior to trapping, the practitioner then uses a cellular-enabled camera that relays images of sounder activity in the trap in real time and also allows the practitioner to trigger the gate closure mechanism only when all members of the sounder have entered the trap.

Despite the obvious advantage of removing an entire sounder at once using whole-sounder traps, the potential for this strategy to improve efficacy of pig control depends heavily on the degree of territoriality among sounders^6^. In the extreme case of no territoriality (extensive space-use sharing with no active defense^7^), multiple sounders are likely to share space, thus dramatically increasing the spatial extent and duration of control efforts required to eradicate pigs from the area. In contrast, if sounders are territorial (defense of space and exclusion of conspecifics), removal of a sounder from an area is more likely to eradicate pigs from that area (aside from lone boars) for some extended period of time, though the duration of this period would depend on the site-fidelity of neighboring sounders and whether they moved into the vacant space^8^. Yet, few studies have examined whether sounders are territorial, as defined by defense of space and exclusion of conspecifics, and those that have examined sounder territoriality yielded contradictory results^9–12^. Sparklin et al.^12^ found that wild pig sounders at Fort Benning Military Reservation in Georgia, USA had nearly exclusive home ranges with little overlap, whereas Boitani et al.^9^ reported near complete overlap among neighboring sounders, although the latter study may have monitored subgroups within the same sounder^11^. Clearly, additional research is warranted to investigate the degree to which sounders are territorial, what factors may alter territoriality among sounders (e.g., anthropogenic food resources, dominant sow characteristics), and consequently, the extent to which whole-sounder trapping can be expected to improve efficacy of control relative to conventional trapping methods^13,14^.

The most commonly used definition of territoriality includes active defense of an area^7^, but some have argued that defense of space is not necessary^15^ and can even be problematic^16^. Moreover, among animals (particularly secretive mammals) for which defense behaviors are not readily observed, many published studies have conceptually defined territoriality to involve exclusive use of space, often evaluated operationally by examining overlap in space use as an index of defense^7^. Unfortunately, no consensus exists on a threshold level of space use overlap that would indicate territoriality. Further, territoriality is recognized, as defined by space use overlap, to exist as points along a continuum, and providing such data points can facilitate comparisons among populations or along gradients of resource conditions^7^. For these reasons, and because exclusive use of space is of greater interest in the context of whole-sounder trapping than the mechanisms by which that space is maintained (including active defense), we herein define territoriality in terms of spatial overlap and assess its degree using indices of spatial overlap.

Our objectives were to characterize the degree of territoriality among wild pig sounders over various durations of time and to assess whether metrics characterizing territoriality (i.e., extent of space-use sharing and frequency of dynamic interactions) were influenced by access to a concentrated and super-abundant food resource in the form of a large municipal-waste landfill. We considered time periods of various duration in case sounders use exclusive space only for short periods. We also evaluated the influence on degree of territoriality of other variables known to affect wild pig movement, including sow and sounder characteristics, accessibility of another preferred habitat, and weather conditions.

## Materials and methods

### Study area

**Figure 1:**
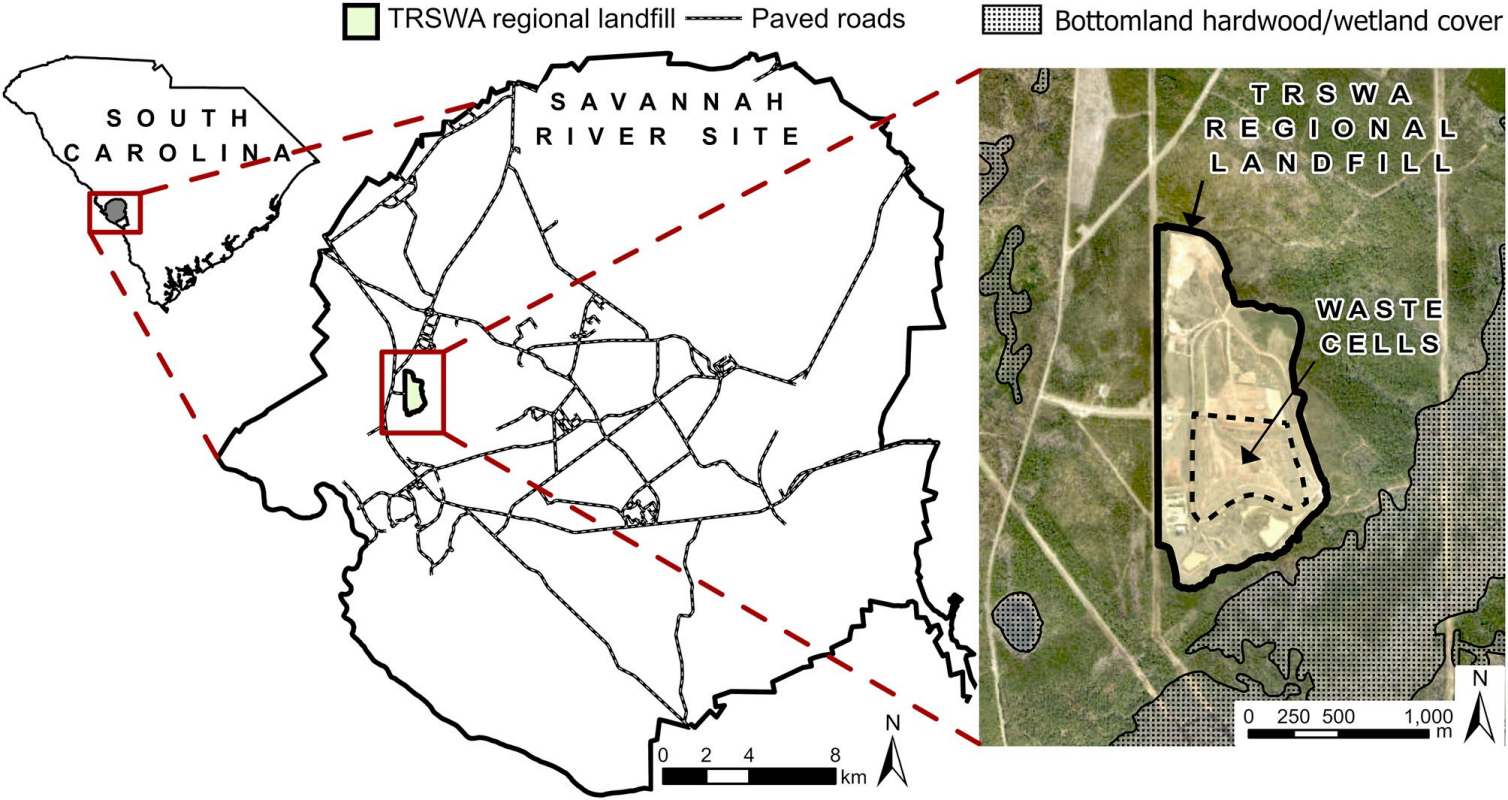
Location of the Three Rivers Solid Waste Authority (TRSWA) regional landfill and waste cells on the Savannah River Site, South Carolina, USA. Map created using ArcGIS Pro version 2.8.1 (https://www.esri.com)^17^.

We conducted the study on the Savannah River Site (SRS; Fig. 1), a 78,000-ha National Environmental Research Park owned and operated by the U.S. Department of Energy in the Upper Coastal Plain of South Carolina, USA. Mean annual temperature is 18º C and mean annual rainfall is 122.5 cm. Approximately 5% of the SRS area was occupied by industrial facilities, with the remainder in managed forest, natural areas, and wetlands. Uplands were dominated by loblolly (*Pinus taeda*) and longleaf (*P. palustris*) pine managed by the U.S. Forest Service on 50–120-year rotations, depending on species and site-specific objectives, and floodplains of the Savannah River and major tributaries were occupied by bottomland hardwood and cypress (*Taxodium distichum*)-tupelo (*Nyssa aquatic* and *N. sylvatica* var. *biflora*) forests.

Wild pigs have been known to exist on SRS since 1950 but likely originated from free-ranging domestic pigs that went feral as early as the eighteenth century^18^. In the 1970s, illegally introduced pigs with wild boar characteristics appeared, so during our study the population consisted of wild boar × feral pig hybrids^18^. Control methods, implemented sporadically since 1952 but more intensively since 1985, include trapping, hunting with dogs, and shooting. Total annual removal during 2014–2016 averaged 1,419 pigs.

Since 1998 the Three Rivers Solid Waste Authority has operated a 9-county regional landfill located on a 134-ha portion of SRS (Fig. 1). Since operations began, the landfill receives about 907 metric tons of waste per day (226,796 metric tons/year), including food waste, household garbage, and other materials. The active waste cells receiving refuse are embedded within the larger footprint of the landfill’s cleared space, which includes grassy fields, open ground, retention ponds, and facilities (Fig. 1). Wild pigs use the landfill extensively, and body mass, litter size, and the number of pig-vehicle collisions are all greater in the vicinity of the landfill than on the rest of SRS^19^.

### Pig density estimation

To provide context to our pig space use data, we used baited camera-trap surveys to estimate pig density during May 2014 and March 2016 in the area surrounding the landfill and encompassing home ranges of GPS-monitored pigs. We deployed 50 white-flash cameras at 500-m spacing in 2014 and 45 cameras at 750-m spacing in 2016 and recorded pig detections over 10 24-h sampling occasions each year. From images, we assigned individual identification to as many pigs as possible based on natural markings (e.g., pelage color and pattern, scars, etc.) and ear tags. We used spatial-capture-recapture^20^ models for partially marked populations to estimate density (Supplementary Methods).

### Wild pig capture and telemetry

During the winters and springs (Nov–Mar) of 2013–2014 and 2015–2016, we used primarily whole-sounder strategies to trap wild pigs throughout an area extending approximately 1.5 km from the landfill boundary (Supplementary Methods). For 2 sounders using areas too remote to install a trap, we captured an adult sow over bait via dart rifle from a tree stand (Supplementary Methods). Because we expected the home range of the dominant mature sow in each sounder to represent that of the sounder, we attached a GPS collar to the largest sow in each sounder, assuming she was the most dominant member. All GPS-collared sows were either subadult (1–3 years) or adult (> 3 years). We programmed collars to acquire GPS fixes every 2 h. Capture and handling procedures were approved by the University of Georgia’s Institutional Animal Care and Use Committee (IACUC protocol numbers A2012 08-004 and A2015 05-004) and were conducted in compliance with the ARRIVE guidelines for the immobilization of animals for studies conducted in the field.

### Space-use estimation

We used dynamic Brownian bridge movement models (dBBMM^21^) to estimate season-long (3.5-month period; 27 Mar to 12 Jul during each year), monthly, and weekly utilization distributions (UD) for each collared sow. Although we tracked pigs outside of the season-long date range, we selected that date range because it maximized sample sizes while maintaining a consistent season-long date range between years. The monthly time period in 2014 included Apr, May, and Jun, and in 2016 included Feb, Mar, Apr, May, Jun, and Jul. The weekly time period in 2014 included 14 7-day periods between 27 Mar and 12 Jul, and in 2016 included 26 7-day periods between 22 Jan and 31 Jul. We specified a window size of 29 and a margin of 11 when estimating dBBMMs based on the temporal resolution of GPS locations and our a priori assumptions about the temporal scale of major behavioral shifts^22^. Based on recent research reporting average GPS collar location fix errors of 10–20 m on similar study sites^23^, we used an error estimate of 20 m when estimating all UDs. We estimated all UDs on identical 50-m resolution spatial grids that encompassed all GPS locations collected during each of 2014 and 2016. We defined home ranges and core areas using 95% and 50% UD isopleths, respectively^24^.

We estimated spatial overlap of home ranges and core areas for each pair (dyad) of neighboring sows across each of our temporal scales. We considered sows to be neighbors if their home range boundaries intersected during any time period^25^. For each dyad, we calculated two- and three-dimensional overlap as the proportional area of overlap of the two UDs (2D) and the volume of intersection (VI), respectively^26^. We estimated dBBMMs and calculated 2D overlap and VI in the R statistical environment (Supplementary Methods^27^).

### Modeling UD size and overlap

We used regression methods to quantify effects of various predictors on UD size and overlap at each of our temporal scales. Given 2D overlap and VI metrics are bound between 0 and 1, we used beta regression and generalized linear mixed-effects models with a beta error distribution. For each of our three response variables (i.e., UD size, 2D overlap, and VI), we developed individual models for each time period to focus our analysis on variation within time periods. We used generalized linear regression rather than mixed-models to analyze season-long UD size and overlap because we lacked repeated season-long measures for dyads. We fit sow ID and sample period (i.e., the specific week or month during weekly or monthly time periods) as random intercepts in each mixed model to account for unbalanced sample sizes and repeated sampling of individual sows^28^. Given VI estimates relate to dyads and that sows could be included in multiple dyads during a given sample period, we used the mean of all VI estimates for dyads including a given sow during each sample period as the response variable in VI models. For example, if a sow was included in four different dyads during a given week, we calculated the sow-specific VI as the mean of the four weekly dyads including the sow. Similarly, we modeled 2D overlap as the mean of sow-specific 2D overlap estimates for dyads including a given sow during each sample period.

We sought to understand the influence of the landfill while accounting for other variables known to affect space use by wild pigs on our study site (e.g.^29^). Hence, for UD size, 2D overlap, and VI, we developed candidate models based on four covariate groups representing effects related to: (1) the landfill; (2) sow characteristics; (3) vegetation cover type; and (4) weather conditions (Supplementary Table S1, Supplementary Methods). The landfill covariate group included distance to the active waste cells within the landfill (m; Dist.WC) and percent of the UD within the landfill boundary (%UD.LF). Sow covariates included body mass (kg; Mass), age (adult or subadult; Age), and sounder size (total number of pigs of all ages in the group; Sounder). Vegetation cover type covariates included the percent of the UD within bottomland hardwood/wetland cover (%UD.BLHW), and weather covariates included period-specific mean temperature (°C; Temp) and barometric pressure (mb; Press).

We included combinations of and interactions among covariates in the four covariate groups, but we limited interactions to the third order to minimize the risk of overparameterizing models and to simplify interpretation. We fit UD level (home range and core area; UD.lev) and year (2014 and 2016; Year) as categorical variables in season-long, monthly, and weekly overlap models, but dropped year from season-long UD size models due to model convergence issues. We did not fit weather covariates in season-long models due to model convergence issues. Additionally, we did not fit interactions involving year or weather covariates in any models to minimize the risk of overparameterizing models. We used second-order Akaike’s Information Criterion (AIC_*c*_^30^) for model selection and considered models with ΔAIC_*c*_ < 2 as plausible models. We estimated regression models in the R statistical environment (Supplementary Methods).

### Dynamic interactions

We examined attraction and avoidance within dyads during each time period using two indices of dynamic interaction that explicitly focus on locations for a dyad that are simultaneous in both space and time^31^. We considered locations of a dyad to be simultaneous in space and time if they were < 50 m and < 10 min apart, respectively. Accordingly, we specified distance and time thresholds of 50 m and 10 min, respectively, in calculation of both dynamic interaction indices. Following Benhamou et al.^32^, we calculated dynamic interaction indices only for dyads with VI estimates > 0.1 during a given time period to avoid calculating indices for dyads with little to no probability of overlapping.

First, we used proximity analysis to estimate the frequency at which pigs of a given dyad were close in time and space^31^, which is calculated as the ratio of locations simultaneous in space and time to locations simultaneous only in time. Second, we calculated the half-weight association index (HAI^33^) to test for attraction or avoidance between individual sows of a given dyad during periods of simultaneous use within a shared area. The HAI index is a localized metric that compares the number of dyad locations simultaneous in space and time within a shared area against the number of solitary locations within a shared area^31^. The HAI is bound between 0 and 1, with values approaching 0 and 1 indicating avoidance or attraction, respectively, within the shared area. Because our study was focused on effects of the landfill resource, we calculated HAI using shared areas defined by polygons for: (1) the landfill boundary; and (2) the waste cells within the landfill boundary (Fig. 1). We estimated dynamic interaction indices in the R statistical environment^27^ (Supplementary Methods).

## Results

### Pig density

Estimated pig density was greater in 2014 (mean density = 20.2 pigs/km^2^; 95% confidence interval [CI] = 17.1–23.2) than in 2016 (mean density = 6.6 pigs/km^2^; 95% CI = 6.5–6.7). Pig density varied spatially during each year, although during both years pig density tended to be higher closer to the landfill and waste cells (Supplementary Fig. S1).

### Wild pig capture and telemetry

We captured most members of 18 target sounders (*n*_2014_ = 7, *n*_2016_ = 11, total pigs captured = 127), except members of the 2 sounders from which sows were darted over bait, and we tracked one sow from each sounder. These 18 sows provided 19,508 GPS locations (*n*_2014_ = 4723, *n*_2016_ = 14,785). Mean sounder size during 2014 and 2016 was 8.8 ± 2.6 (SD) and 6.4 ± 6.5 pigs, respectively, and mean mass of tracked sows during 2014 and 2016 was 81.8 ± 19.1 and 72.5 ± 19.8 kg, respectively. Of the 7 sows tracked in 2014, 4 were subadult and 3 were adult, and of the 11 sows tracked in 2016, 4 were subadult and 7 were adult.

### Space-use

**Figure 2:**
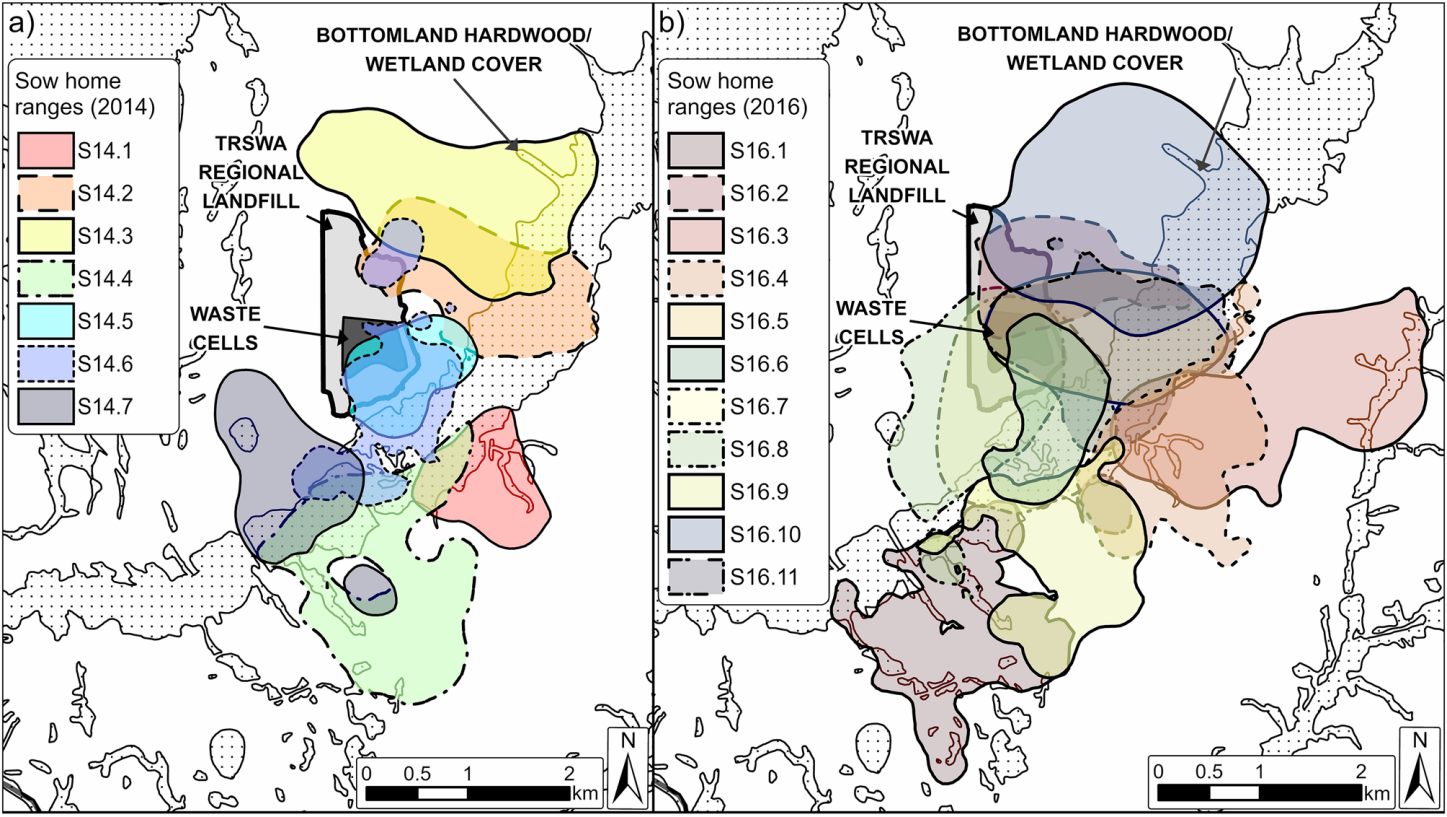
Season-long (3.5 months; 27 Mar–12 Jul) home ranges (95% utilization distribution contours) of wild pigs (*Sus scrofa*) tracked on Savannah River Site, South Carolina, USA, in 2014 (**a**; *n*_*pigs*_ = 7) and 2016 (**b**; *n*_*pigs*_ = 11). Map created using ArcGIS Pro version 2.8.1 (https://www.esri.com)^17^.

Mean home range and core area sizes were largest during the season-long time period and declined during shorter monthly and weekly time periods (Table 1). We identified 19 and 30 dyads in 2014 and 2016, respectively. At the season-long scale, dyads exhibited considerable 2D overlap (Fig. 2), but average VI estimates were generally low across all time periods. Overall, mean 2D overlap and VI of home ranges were < 0.3 and < 0.1, respectively, across all time periods, and mean 2D overlap and VI of core areas were < 0.2 and < 0.05, respectively, across all time periods (Supplementary Figs. S2 and S3). Of the 19 and 30 dyads identified in 2014 and 2016, respectively, 5 dyads from each year had VI estimates < 0.1 across time periods.

**Table 1:** Mean and standard deviation (SD) of wild pig (*Sus scrofa*) home range and core area sizes (hectare) across time periods (Season-long [*n*_*season*_ = 2; *n*_*pigs*_ = 14], Month [*n*_*months*_ = 6; *n*_*pigs*_ = 18], and Week [*n*_*weeks*_ = 26; *n*_*pigs*_ = 17]) and utilization distribution (UD) levels representing home ranges (95% UD contour; HR) and core areas (50% UD contour; CA) for pigs tracked on the Savannah River Site, South Carolina, USA in 2014 and 2016.

Time period	UD level	2014	2016
		Mean ± SD	Mean ± SD
Season-long	HR	216.3 ± 98.5	276.7 ± 103.6
	CA	33.1 ± 17.4	43.4 ± 20.7
Month	HR	206.0 ± 98.7	241.8 ± 105.8
	CA	30.4 ± 20.4	39.9 ± 19.6
Week	HR	166.0 ± 100.3	202.1 ± 119.3
	CA	25.0 ± 18.7	37.0 ± 23.2

### Modeling UD size and overlap

**Figure 3:**
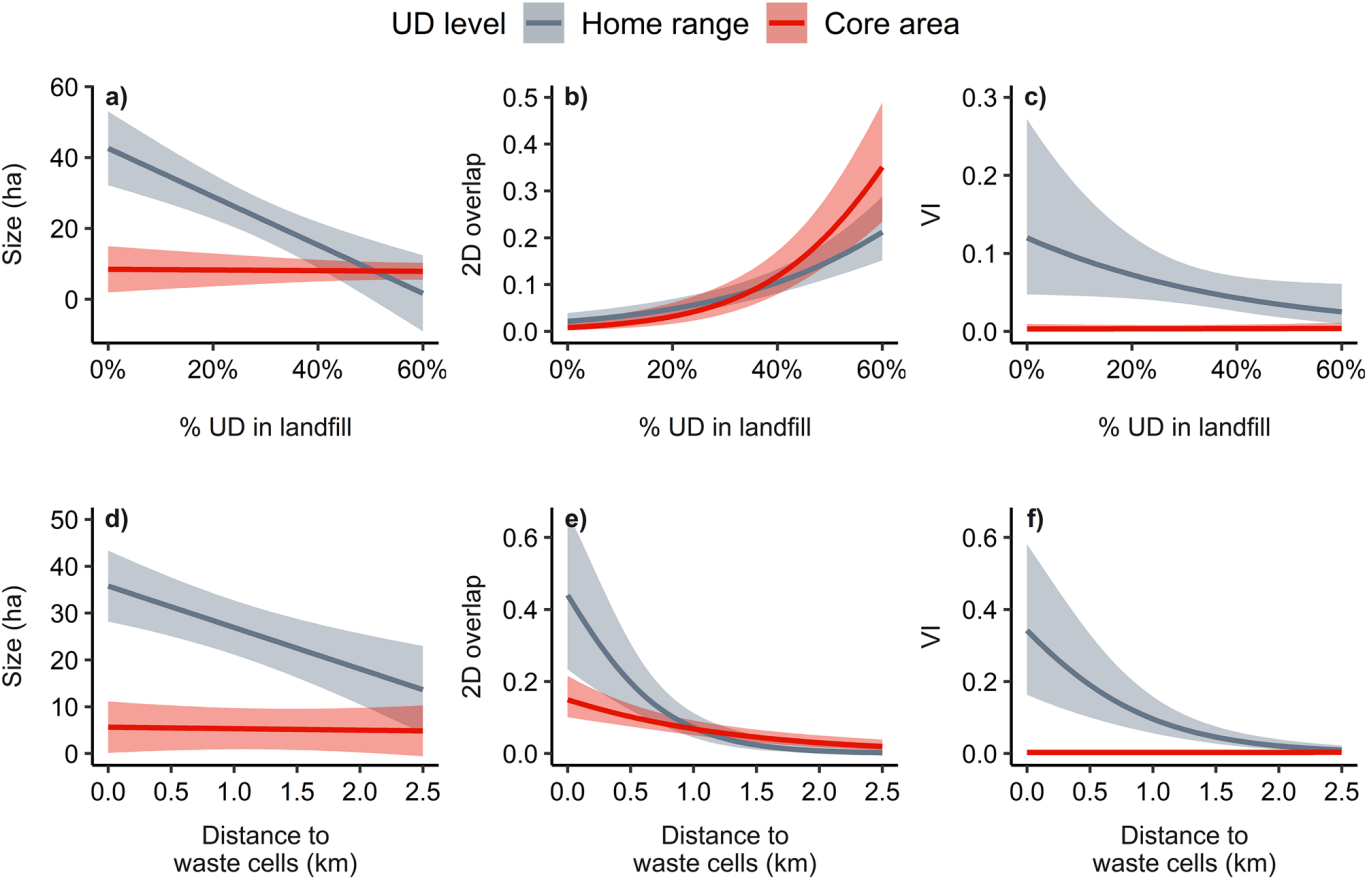
Predicted effects of percentage of utilization distribution (UD) in the landfill (% UD in landfill) and distance from UD boundaries to the waste cells within the landfill (Distance to waste cell [km]) on UD size (ha; **a** and **d**), proportional area of overlap (2D overlap; **b** and **e**), and volume of intersection (VI; **c** and **f**) of season-long (*n* = 2) UDs for wild pigs (*Sus scrofa*; *n* = 14) on Savannah River Site, South Carolina, USA, in 2014 and 2016. Figure created in the R statistical environment version 4.0.5 (https://www.cran.r-project.org)^27^.

The top season-long UD size model included UD level, landfill, vegetation cover type, and sow covariates and an interaction between UD level and percentage of UD in the landfill (Supplementary Table S2). Overall, season-long UD size at the home range level increased as distance to the active waste cells decreased (Fig. 3d), and season-long UD size decreased for sows with home ranges that included a greater percentage of the landfill (Fig. 3a; Supplementary Table S3). The top monthly and weekly UD size models included interactions among UD level, landfill, sow, and vegetation cover type covariates (Supplementary Tables S4 and S5). Monthly and weekly UD size increased for sows with UDs that were closer to the active waste cells (Supplementary Fig. S4a.i, a.ii) and decreased for sows with UDs that included a greater percentage of the landfill (Supplementary Fig. S5a.i, a.ii).

The top season-long 2D overlap model included interactions among UD level, landfill, and sow covariates (Supplementary Table S6). Overall, season-long 2D overlap increased for sows with UDs that were closer to the waste cells (Fig. 3e) and for sows with UDs that included a greater percentage of the landfill (Fig. 3b). At the home range level, season-long 2D overlap decreased for older sows but increased for heavier sows (Table 2). The top monthly 2D overlap model included interactions between UD level and landfill covariates (Supplementary Table S7). Monthly 2D overlap increased for sows with UDs that were closer to the waste cells (Supplementary Fig. S4b.i) and for sows with home-ranges that included a greater percentage of the landfill (Supplementary Fig. S5b.i; Table 2). The top weekly 2D overlap model included interactions among UD level, landfill, sow, cover type, and weather covariates (Supplementary Table S8). Overall, weekly 2D overlap increased for sows with UDs that were closer to the waste cells (Supplementary Fig. S4b.ii), that included a greater percentage of the landfill (Supplementary Fig. S5b.ii), for heavier sows, and for sub-adult sows (Table 2).

**Table 2:** Odds ratios (OR) and 90% confidence intervals (CI) for the top supported model of two-dimensional utilization distribution (UD) overlap, estimated as the proportional area of UD overlap, for wild pigs (*Sus scrofa*) across time periods (Season-long [*n*_*season*_ = 2; *n*_*pigs*_ = 14], Month [*n*_*months*_ = 6; *n*_*pigs*_ = 18], and Week [*n*_*weeks*_ = 26; *n*_*pigs*_ = 17]) tracked on Savannah River Site, South Carolina, USA during 2014 and 2016. Colons separating covariate names denote interaction terms and asterisks denote 90% CI of odds ratios that did not overlap 1.

Predictors^a^	Season-long	Month	Week
	OR (90% CI)	OR (90% CI)	OR (90% CI)
Year	3.60* (2.22–5.84)	1.23 (0.78–1.95)	2.41* (1.74–3.32)
UD.lev	0.01* (0.00–0.09)	1.01 (0.66–1.54)	0.80 (0.36–1.76)
Dist.WC	0.22* (0.09–0.56)	0.81* (0.68–0.97)	0.86* (0.75–0.98)
%UD.LF	1.52* (1.32–1.75)	1.21* (1.07–1.36)	1.10* (1.01–1.19)
Age	1.19 (0.83–1.70)		1.64* (1.18–2.28)
Mass	0.92 (0.82–1.02)		1.13* (1.05–1.22)
Sounder	1.04 (0.98–1.10)		1.01 (0.98–1.04)
%UD.BLHW	0.91 (0.82–1.02)		0.93* (0.87–0.99)
Temp			1.04* (1.03–1.06)
Press			1.00 (0.98–1.03)
UD.lev: Dist.WC	0.19 (0.02–1.54)	0.90 (0.65–1.24)	1.00 (0.84–1.20)
UD.lev: %UD.LF	1.32* (1.05–1.68)	0.75* (0.64–0.88)	0.89* (0.80–0.97)
UD.lev: Age	0.21* (0.06–0.70)		1.04 (0.71–1.53)
UD.lev: Mass	1.53* (1.13–2.08)		0.99 (0.92–1.07)
UD.lev: %UD.BLHW			1.07 (0.99–1.14)
UD.lev: Sounder	1.12 (0.95–1.33)		1.00 (0.98–1.03)
Dist.WC: %UD.LF	1.10 (0.94–1.29)		
Dist.WC: Age	2.46* (1.38–4.39)		
Dist.WC: Mass	1.06 (0.94–1.19)		
Dist.WC: Sounder	0.97 (0.90–1.05)		
UD.lev: Dist.WC: %UD.LF	0.43* (0.30–0.60)		
UD.lev: Dist.WC: Age	6.14* (2.45–14.9)		
UD.lev: Dist.WC: Mass	1.34 (0.96–1.87)		
UD.lev: Dist.WC: Sounder	0.81 (0.63–1.06)		

^a^UD.lev = utilization distribution (UD) contour level (i.e., 95% and 50% contours representing home ranges and core areas, respectively); Sounder = Sounder size; Mass = body mass (kg); Temp = temperature (C°); Press = barometric pressure (mb); HA.UD.LF = hectare (ha) of UDs within the landfill boundary; %UD.LF = percent of UDs within the landfill; HA.UD.BLHW = ha of UDs within bottomland hardwood/wetland cover; %UD.BLHW = percent of UDs within bottomland hardwoods/wetland cover; and Dist.WC = distance from UD boundaries to the waste cells within the landfill (km).

Top season-long, monthly, and weekly VI models included interactions among UD level, landfill, sow, and vegetation cover type covariates (Supplementary Tables S9-11). Season-long, monthly, and weekly VI increased for sows with home ranges that were closer to the waste cells and decreased for sows with home ranges that included a greater percentage of the landfill (Fig. 3c,f, Supplementary Figs. S4c.i and c.ii, and S5c.i and c.ii). Additionally, season-long and weekly VI decreased for sows with home ranges that included a greater percentage of bottomland hardwood/wetland cover and for heavier sows (Table 3). Season-long VI increased at the home range level for sows in larger sounders, but decreased for older, heavier sows.

**Table 3:** Odds ratio estimates (OR) and 90% confidence intervals (90% CI) for the top supported model of three-dimensional overlap of utilization distributions, estimated as the volume of intersection, for wild pigs (*Sus scrofa*) across time periods (Season-long [*n*_*season*_ = 2; *n*_*pigs*_ = 14], Month [*n*_*months*_ = 6; *n*_*pigs*_ = 18], and Week [*n*_*weeks*_ = 26; *n*_*pigs*_ = 17]) tracked on Savannah River Site, South Carolina, USA during 2014 and 2016. Colons separating predictor names denote interaction terms and asterisks denote 90% CI of odds ratios that did not overlap 1.

Predictors^a^	Season-long	Month	Week
	OR (90% CI)	OR (90% CI)	OR (90% CI)
Year	1.54 (0.71–3.35)	1.03 (0.56–1.91)	0.91 (0.69–1.21)
UD.lev	9.9* (7.9–21.7)	8.1* (3.1–22.6)	8.5* (3.0–24.0)
Dist.WC	1.01 (0.62–1.66)	1.09 (0.80–1.48)	0.93 (0.79–1.11)
%UD.LF	1.41 (0.14–14.2)	1.28 (0.29–5.64)	0.77 (0.45–1.31)
Age	0.54 (0.16–1.84)	1.11 (0.58–2.13)	1.05 (0.65–1.67)
Mass	0.89 (0.69–1.16)	0.98 (0.86–1.13)	0.98 (0.89–1.06)
Sounder	1.05 (0.95–1.17)	1.01 (0.96–1.06)	1.00 (0.97–1.04)
%UD.BLHW	0.08 (0.00–1.81)		0.76 (0.36–1.61)
UD.lev: Dist.WC	0.20* (0.09–0.44)	0.62* (0.43–0.90)	0.64* (0.52–0.79)
UD.lev: %UD.LF	0.04 (0.00–1.37)	0.08* (0.01–0.50)	0.21* (0.08–0.51)
UD.lev: Age	0.11* (0.03–0.46)	1.45 (0.65–3.22)	1.21 (0.75–1.94)
UD.lev: Mass	0.55* (0.40–0.77)	0.86 (0.72–1.03)	0.89* (0.81–0.99)
UD.lev: Sounder	1.28* (1.11–1.46)	1.01 (0.95–1.07)	1.02 (0.98–1.05)
UD.lev: %UD.BLHW	0.00* (0.00–0.02)		0.39* (0.15–0.99)

^a^UD.lev = utilization distribution (UD) contour level (i.e., 95% and 50% contours representing home ranges and core areas, respectively); Sounder = Sounder size; Mass = body mass (kg); Temp = temperature (C°); Press = barometric pressure (mb); HA.UD.LF = hectare (ha) of UDs within the landfill boundary; %UD.LF = percent of UDs within the landfill; HA.UD.BLHW = ha of UDs within bottomland hardwoods; %UD.BLHW = percent of UDs within bottomland hardwoods/wetland cover; and Dist.WC = distance from UD boundaries to the waste cells within the landfill (km).

### Dynamic interactions

We estimated dynamic interaction indices for 14 and 25 dyads that had VI estimates > 0.1 during any time period in 2014 and 2016, respectively. Overall, dynamic interaction indices were generally low across all time periods during 2014 and 2016 (Table 4). Proximity analyses indicated dyads from each of 2014 and 2016 were rarely close in space and time during any time period (Supplementary Fig. S6, Table 4). In 2014, pigs from only two dyads were close in space and time across all time periods, mean landfill-HAI estimates were < 0.0001 across all time periods, and mean waste-cell-HAI estimates were all 0 (Table 4). In 2016, mean HAI estimates were all < 0.031 across time periods (Supplementary Fig. S7, Table 4).

**Table 4:** Mean and standard deviation (SD) of frequency of wild pig (*Sus scrofa*) locations that were within 10 min and 50 m apart (Prox) and half-weight association (HAI) indices during periods of simultaneous use within the landfill footprint (HAI [Landfill]) and the waste cells within the landfill footprint (HAI [Waste cell]) across time periods (Season-long [*n*_*season*_ = 2; *n*_*pigs*_ = 14], Month [*n*_*months*_ = 6; *n*_*pigs*_ = 18], Week [*n*_*weeks*_ = 26; *n*_*pigs*_ = 17]) for pigs tracked on the Savannah River Site, South Carolina, USA, in 2014 and 2016.

Year	Index	Season-long	Month	Week
		Mean ± SD	Mean ± SD	Mean ± SD
2014	Prox	0.010 ± 0.009	0.025 ± 0.014	0.047 ± 0.030
	HAI [Landfill]	0	0	0
	HAI [Waste cell]	0	0	0
2016	Prox	0.010 ± 0.007	0.011 ± 0.007	0.009 ± 0.012
	HAI [Landfill]	0.009 ± 0.012	0.031 ± 0.021	0.009 ± 0.006
	HAI [Waste cell]	0.015 ± 0.008	0.024 ± 0.018	0.013 ± 0.003

## Discussion

Under a strict definition of territoriality as involving exclusive use of an area^34^, wild pig sounders in our study cannot be considered territorial. Our dyads often exhibited extensive overlap in space at the level of the home range, and to a lesser extent, even core areas. However, some researchers have recognized the degree of territoriality is characterized by a continuum, which often has been evaluated using the extent to which individual home ranges overlap, although the extent of overlap that distinguishes territorial from non-territorial behavior is unclear (reviewed by^7^).

Aspects of space use sharing other than simple 2D spatial overlap (e.g., VI, frequency of simultaneous locations within shared areas) can also inform our understanding of the degree of territoriality in a species. By considering these other metrics, we detected clear signs of among-sounder territoriality that were not adequately captured by area-based estimates of 2D overlap. Although VI estimates for dyads were often > 0, even in core areas, mean values were nevertheless quite low, indicating very low probability that dyads shared use of the same space. Similarly low values of VI have been reported for other species known to be territorial (e.g., American redstart, *Setophaga ruticilla*^35^). Additionally, negligible but non-zero VI estimates can occur even in strictly territorial species, because UDs inherently include peripheral areas beyond the extent of dyad locations^36^. Much of the 2D overlap we observed likely reflects occasional excursions into a neighbor's home range, which typically is not frequent among territorial species and can misrepresent the degree of territoriality, as judged by the extent of space-use sharing^26^. The VI metric on the other hand is based on the intensity of use within shared areas, which reduces the influence of occasional excursions and reflects more nuanced aspects of the extent to which neighboring individuals share space. Finally, our dynamic interaction metrics demonstrated that sounders avoided each other, very rarely occurring in the same place at the same time. Likewise, low direct contact rates for members of neighboring sounders have recently been reported from other studies in the southeastern U.S.^37,38^, although Yang et al.^38^ documented some direct inter-sounder contacts. We therefore believe that despite sharing as much as 60% of the area of their home ranges with neighboring sounders, our results largely support the conclusions of Gabor et al.^11^ and Sparklin et al.^12^ in that wild pig sounders displayed territorial characteristics by generally avoiding each other in both space and time.

The degree of territoriality we observed among sounders apparently was influenced to some extent by the tremendous food resource provided by the landfill. Our best-supported VI model indicated the intensity of use within shared areas of home ranges increased for dyads that were closer to the waste cells. This suggests that although sounders rarely came in contact at the waste cells, they nevertheless shared space to a greater extent closer to the waste cells than away from them. In contrast, the intensity of use within shared areas declined with increases in both the percentage of the landfill footprint and of bottomland hardwoods in home ranges. Sounders frequently were located within the landfill footprint during nights they were never observed accessing the waste cells. Wild pigs have been observed foraging extensively in the open fields within the landfill footprint but outside of the waste cells (K. Cox, U.S. Forest Service, personal communication), suggesting these landfill fields also represent a valuable resource for wild pigs, if perhaps not to the extent of the concentrated resources in the waste cells themselves. Given the similar decline in VI with increases in percentage of the UD in the landfill and in bottomland hardwood/wetland forests, our results suggest some preferred natural habitats (i.e., bottomland hardwood and wetland forests^23^) may be of comparable value to the resources in the landfill fields. Thus, the food waste available to pigs within the waste cells represents a very high value resource^19^, but the landfill fields and bottomland hardwoods are also important resources, likely intermediate in value between the waste cells and less desirable habitats. Such relative differences in resource levels between the waste cells and these intermediate-value habitats may explain opposing effects of these variables on the intensity of use within shared areas. The decrease in VI as percentage of UD in the landfill and bottomland hardwoods increased indicates sounders were more territorial within these habitats, possibly because the benefits of maintaining exclusive access to resources at intermediate levels outweigh the costs of territorial defense. In contrast, the increase in VI closer to the waste cells suggests resources within the waste cells were so abundant as to not be worth the cost of defense^39^.

Home range sizes were generally smaller and overlapped less when they included a greater percentage of the landfill, suggesting a mechanism by which more territories could be supported in the vicinity of the landfill by the resources provided there. Similarly, resource-mediated territory packing has been shown to be facilitated by reduced home range size in other taxa^40^. However, sizes of home ranges increased when they were closer to the waste cells, contrasting with the expectation that increased resource availability would lead to decreased home range sizes. We are uncertain as to an explanation for this apparent contradiction in the effect of landfill and waste cell resources on home range size but suspect it may have been attributable to the spatial distribution of cover types within home ranges. Sounders with home ranges closer to the waste cells exploited the concentration of food resources there, which were abundant to the point defense of space and resources was rendered unnecessary. However, that these sounders did not need to defend space near the waste cell to maintain access to food may have reduced costs associated with expanding their home ranges to access other resources (cover and water), despite being closer to concentrated food resources near waste cells. In contrast, sounders used less space and were more territorial when home ranges were located where the spatial configuration of cover types enabled sounders to reduce home range sizes while still maintaining access to high quality food resources in the landfill and critical food, cover, and water resources in bottomland hardwoods. Thus, sounders with home ranges that included a greater percentage of the landfill, but not necessarily home ranges closer to the waste cells, used less space because their home ranges were positioned such that they could reduce home range size while still maintaining largely exclusive access to resources in both the landfill and bottomland hardwood/wetland cover.

Although we did not explicitly assess the effects of pig density on territoriality, the difference in density between 2014 and 2016 may have influenced space use of pigs around the landfill. We estimated a 67% reduction in density between 2014 and 2016. This marked reduction apparently was due to particularly intensive control efforts that occurred in the 32.4-km^2^ administrative unit surrounding the landfill during the 21 months between our camera surveys in 2014 and 2016. From May 2014 through February 2016, 474 pigs were removed (14.6/km^2^; U.S. Forest Service, unpublished data). The mean percentage of the UDs in the landfill was greater during 2016 (33.9%) than 2014 (8.7%), indicating greater usage of the landfill in 2016, and season-long home range size was somewhat larger in 2016 (276.7 ha) than 2014 (216.3 ha), both relationships potentially facilitated by reduced density that lowered competition across the study area in 2016 compared to 2014.

A potential explanation of the level of spatial overlap we observed may pertain to sounder structure. Unlike Gabor et al.^11^ and Sparklin et al.^12^, Boitani et al.^9^ reported complete overlap of two sounders of wild boar in Italy. Gabor et al.^11^ suggested that the apparently conflicting results between their study and that of Boitani et al.^9^ were attributable to differing definitions of sounders between the two studies. Gabor et al.^11^ considered sounders as stable groups of breeding-aged females and their young that shared a common range and often form non-random subgroups. They proposed that the female groups monitored by Boitani et al.^9^ may have corresponded more closely to what Gabor et al.^11^ considered subgroups of the same sounder. Like Boitani et al.^9^, we lacked genetic information on relatedness of various sounders in our study area and may therefore have monitored subgroups as separate sounders, when in fact they belonged to the same sounder. Had that been the case, however, we would have expected to observe greater VI and dynamic interaction values, as such subgroups would not likely avoid areas of shared space and would not avoid each other.

Effects of sow characteristics on overlap metrics were equivocal. Older and heavier sows, presumably dominant in interactions between sounders, had lower mean VI and older sows had lower mean 2D spatial overlap, indicating that sounders with such sows had a competitive advantage that enabled them to exclude other sounders with younger and smaller sows. Heavier sows also had greater 2D spatial overlap with neighbors, suggesting their larger size conferred a competitive advantage that lowered perceived risks of intruding into home ranges of neighboring sounders. However, contrary to expectations, larger sounders, presumably dominant to smaller sounders, had higher VI. Our results suggest increased sounder size does not necessarily confer a competitive advantage in wild pigs, in contrast to many other social species^41–43^. This finding could have been driven by differences in age composition among our sounders. For instance, a sounder with a single adult sow and 8 piglets would likely be a weaker competitor than a sounder with 4 adult sows and 2 yearlings, though the former is the larger sounder. However, given the generally low VI estimates across all sounders, we suspect that these apparent effects of sow characteristics may be confounded by other aspects of sounder structure (e.g., age and weight of all pigs in a given sounder, relatedness among neighboring sounders). Further research is needed to determine what aspects of individual sows and sounders are most influential on sounder territoriality.

Collectively, our findings have implications for programs aimed at control of this invasive species based on removal of entire sounders. One tenet of whole-sounder trapping assumes that sounders are territorial and have high site fidelity, the implication of which is that when a sounder is removed, pigs (other than lone boars or small bachelor groups) are then absent from that territory for some period of time^6,12^. Monitoring response to an experimental removal, Bastille-Rousseau et al.^8^ reported limited evidence of substantive shifts into the removal area by adjacent GPS-collared pigs during 14 weeks of monitoring post-removal. However, despite the generally territorial behavior we observed among sounders and the fact that sounders avoided each other, our 2D home range data indicated their home range boundaries overlapped extensively in space. Thus, removal of one sounder would not necessarily render that territory free of pigs. Furthermore, several different sounders used the landfill, suggesting a concentrated resource such as a productive crop field with high nutritional value may also be used by multiple sounders. In such cases, where multiple sounders, each from a different territory, converge on a single high-value resource, trapping at or around that location (e.g., along nearby access routes) may effectively remove sounders from a much larger area than a single territory^12^. Indeed, four of our sounders in 2016 were captured at a single trap location within 125 m of the landfill over a period of 9.5 weeks. Thus, managers should not assume that when a single sounder is removed near a concentrated resource, no additional sounders can be captured there until additional monitoring reveals no more are present. However, at the greatest distance from the waste cells, 2D overlap approached zero for our season-long period, suggesting that in the absence of such a resource, additional sounders are not likely to be captured at the same trap location.

We expected that if territoriality among sounders was weak, then evidence of territoriality may only be detected over shorter time periods (e.g., weeks); i.e., the greater the duration, the greater the likelihood occasional incursions by neighbors would obscure exclusivity of space use. Although mean VI values were uniformly low, regardless of our time period duration, the magnitude of landfill predictor effects declined as the duration of time periods shortened. Such attenuation of landfill predictor effects over shorter time periods suggests the degree of territoriality among sounders is strongest over shorter time periods. Nevertheless, our 3.5-month season period is applicable to the length of time during which most wild pig trapping efforts typically occur, often during winter and early spring when availability of natural foods is low and baiting is most effective. Thus, we believe that control operators should expect sounders to tend to behave in a generally territorial manner for the duration of most trapping efforts, but multiple sounders may use the same space in the presence of concentrated high-quality resources, such as those provided by the landfill on SRS or agricultural fields.

## Supplementary Information

Supplementary Information.

## Data Availability

Data available from the Figshare Repository https://figshare.com/s/94b32f0d8a180dfd4979.
