# Supplementary material for: Food resources affect territoriality of invasive wild pig sounders with implications for control
Source: Sci Rep. 2021 Sep 22;11:18821. doi: 10.1038/s41598-021-97798-z (PMC8458451; doi:10.1038/s41598-021-97798-z)
Supplement: Supplementary file 1 — Supplementary Information. [file 41598_2021_97798_MOESM1_ESM.docx]

**SUPPLEMENTARY INFORMATION**

24 August 2021

**Food resources affect territoriality of invasive wild pig sounders with implications for control**

John C. Kilgo^1^*, James E. Garabedian^1^, Mark Vukovich^1,2^, Peter E. Schlichting^3,4^, Michael E. Byrne^3,5^, James C. Beasley^3^

^1^ USDA Forest Service, Southern Research Station, P.O. Box 700, New Ellenton, SC 29809, USA; ^2^ USDA Forest Service, Shawnee National Forest, 602 North First St., Vienna, IL, 62995, USA; ^3^ Savannah River Ecology Laboratory, Warnell School of Forestry and Natural Resources, University of Georgia, P.O. Drawer E, Aiken, SC 29802, USA; ^4^ Illinois Department of Natural Resources, 1 Natural Resources Way, Springfield, IL 62702, USA

* Correspondence: john.kilgo@usda.gov

**Methods:**

**Wild pig density estimation**: We deployed 50 cameras (white-flash, Reconyx, Holmen, WI) at 500-m spacing in 2014 and 45 cameras at 750-m spacing in 2016 and recorded pig detections over 10 24-hour sampling occasions each year. We baited each camera with 12 kg of shelled corn upon deployment and another 12 kg on day 5. From the images, we assigned individual identification to as many pigs as possible based on natural markings (e.g., pelage color and pattern, scars, etc.) and ear tags. We used spatial-capture-recapture (SCR) models for partially marked populations to leverage data on both marked and unmarked individuals when estimating density (hereafter, partially marked SCR^1,2^. We used data augmentation^3,4^ to add detection histories of 0 for *M* individuals present but unobserved in the camera grid. Following Royle et al.^4^, we specified *M* to 3 times the number of marked pigs identified in camera images during each of 2014 (*M* = 867) and 2016 (*M* = 720), such that *M* was larger than the total number of pigs present in the camera grid during each year. We used 1-km buffers around camera grids when fitting partially marked SCR models for both survey years. For each survey year, we ran three chains for 250,000 iterations with a burn-in of 50,000 in R using an MCMC algorithm from the contributed package "scrbook"^5^.

**Wild pig trapping and handling**: We deployed trail cameras baited with shelled corn to attract and identify the composition of wild pig sounders. Once a sounder was conditioned to bait, we erected a trap (JagerPro, Columbus, GA, USA or BoarBuster, W-W Livestock Systems, Thomas, OK, USA) and monitored sounder activity in the trap via cellular-enabled remote camera until the entire sounder entered the trap, at which time we triggered the gate. We immobilized pigs using a combination of Telazol (4.4 mg/kg, Fort Dodge Animal Health, Fort Dodge, IA, USA) and xylaxine hydrochloride (2.2 mg/kg, ZooPharm, Fort Collins, CO, USA) delivered via dart rifle (X-Caliber, PneuDart, Williamsport, PA, USA). For 2 sounders using areas too remote to install a trap, we captured the adult sow over bait via dart rifle from a climbing tree stand (Summit Treestands, LLC, Decatur, AL, USA). For each captured pig, we assigned 1 of 5 age classes (piglet: <8 mo; juvenile: 9–12 mo; yearling: 13–19 mo; subadult: 20–35 mo; and adult: ≥36 mo) based on tooth eruption patterns^6^, recorded sex and body mass, and attached ear tags. We attached a GPS collar (Globalstar Track S or LiteTrack, Lotek Wireless, Inc.) to the largest sow in each sounder, and we programmed collars to acquire GPS fixes every 2 hours. Due to frequent weight fluctuations associated with pregnancy and parturition, we equipped collars with a drop-off mechanism programmed to activate after 8 months.

**R packages used in analysis**: We conducted analyses in the R statistical environment^7^ as follows. We estimated dynamic Brownian Bridge Movement Models and calculated 2D overlap and VI using the contributed packages “adehabitatHR”^8^, “BBMM”^9^, “move”^10^, “rgeos”^11^, and “sp” ^12,13^. We used the base R function “lm” and the contributed package “betareg”^14^ to fit season-long UD size and overlap models, respectively. We used the contributed package “glmmTMB”^15^ to fit monthly, weekly, and daily UD size and overlap models and “ggeffects”^16^ to visualize predictor effects. We estimated dynamic interaction indices using the contributed package “wildlifeDI”^17^.

**Description of predictor variables for overlap analyses**: The landfill predictor group included distance to the active waste cells within the landfill (m, calculated as the distance from the nearest edge of the UD to the nearest edge of the waste cells; Dist.WC), and percent of the UD within the landfill boundary (%UD.LF). Sow covariates included body mass (kg; Mass) and age (adult or subadult; Age^6^), as measured at time of capture; and sounder size (total number of pigs of all ages in the group; Sounder), as determined via trail camera surveillance during trapping and camera-trap surveys during each year. Vegetation cover type covariates included the percent of the UD within bottomland hardwood/wetland cover (%UD.BLHW), and weather covariates included period-specific mean temperature (^o^C; Temp) and barometric pressure (mb; Press), calculated using measurements taken at 15-minute intervals at 2-m above ground-level, obtained from an atmospheric monitoring station 6.7 km from the landfill.

**References**

1. Chandler, R. B. & Royle, J. A. Spatially explicit models for inference about density in unmarked or partially marked populations. *Ann. Appl. Stat.* **7**, 936–954 (2013).

2.   Royle, J. A., Chandler, R. B., Sollmann, R., & Gardner, B. *Spatial Capture-Recapture*. (Academic Press, 2014).

3. Royle, J. A., Karanth, K. U., Gopalaswamy, A. M. & Kumar, N. S. Bayesian inference in camera trapping studies for a class of spatial capture–recapture models. *Ecology* **90**, 3233–3244 (2009).

4. Royle, J. A., Dorazio, R. M. & Link, W. A. Analysis of multinomial models with unknown index using data augmentation. *J. Comput. Graph. Stat.* **16**, 67–85 (2007).

5. Royle, J. A., Chandler, R., Sollmann, R. & Gardner, B. *scrbook: Companion to the book: Spatial Capture Recapture (2014)* (2020).

6. Mayer, J. J. A simple field technique for age determination of adult wild pigs: Environmental information document. *Westinghouse Savannah River Co. WSRC-RP-2002-00635 Westinghouse Savannah River Co. Aiken SC* (2002).

7. R Core Team. *R: A language and environment for statistical computing.* R Foundation for Statistical Computing (2021).

8. Calenge, C. The package adehabitat for the R software: A tool for the analysis of space and habitat use by animals. *Ecol. Model.* **97**, 516–519 (2006).

9. Nielson, R. M., Sawyer, H. & McDonald, T. L. *BBMM: Brownian bridge movement model*. (2013).

10. Kranstauber, B., Smolla, M. & Scharf, A. K. *move: Visualizing and analyzing animal track data*. (2020).

11. Bivand, R. *et al.* *rgeos: Interface to geometry engine - open source ('GEOS’)*. (2020).

12. Pebesma, E. J. & Bivand, R. S. Classes and methods for spatial data in R. *R News* **5**, (2005).

13. Bivand, R., Pebesma, E. J. & Gómez-Rubio, V. *Applied spatial data analysis with R*. (Springer, 2013).

14. Cribari-Neto, F. & Zeileis, A. Beta Regression in R. *J. Stat. Softw.* **34**, 1–24 (2010).

15. Brooks, M. E. *et al.* glmmTMB balances speed and flexibility among packages for zero-inflated generalized linear mixed modeling. *R J.* **9**, 378–400 (2017).

16. Lüdecke, D., Aust, F., Crawley, S. & Ben-Shachar, M. S. *ggeffects: Create tidy data frames of marginal effects for ‘ggplot’ from model outputs*. (2021).

17. Long, J. *wildlifedi: Calculate indices of dynamic interaction for wildlife tracking data*. (2021).

18. ESRI. *ArcGIS Pro.* Environmental Systems Research Institute (2021).

Figure S1. Spatially explicit density of wild pigs (*Sus scrofa*) estimated using a partially marked spatial-capture-recapture model and remote camera data obtained during camera surveys (camera locations indicated by yellow dots) conducted around the Three Rivers Solid Waste Authority landfill (red polygon) on Savannah River Site, South Carolina, USA, in 2014 and 2016. Map created in the R statistical environment version 4.0.5 (www.cran.r-project.org)^7^.


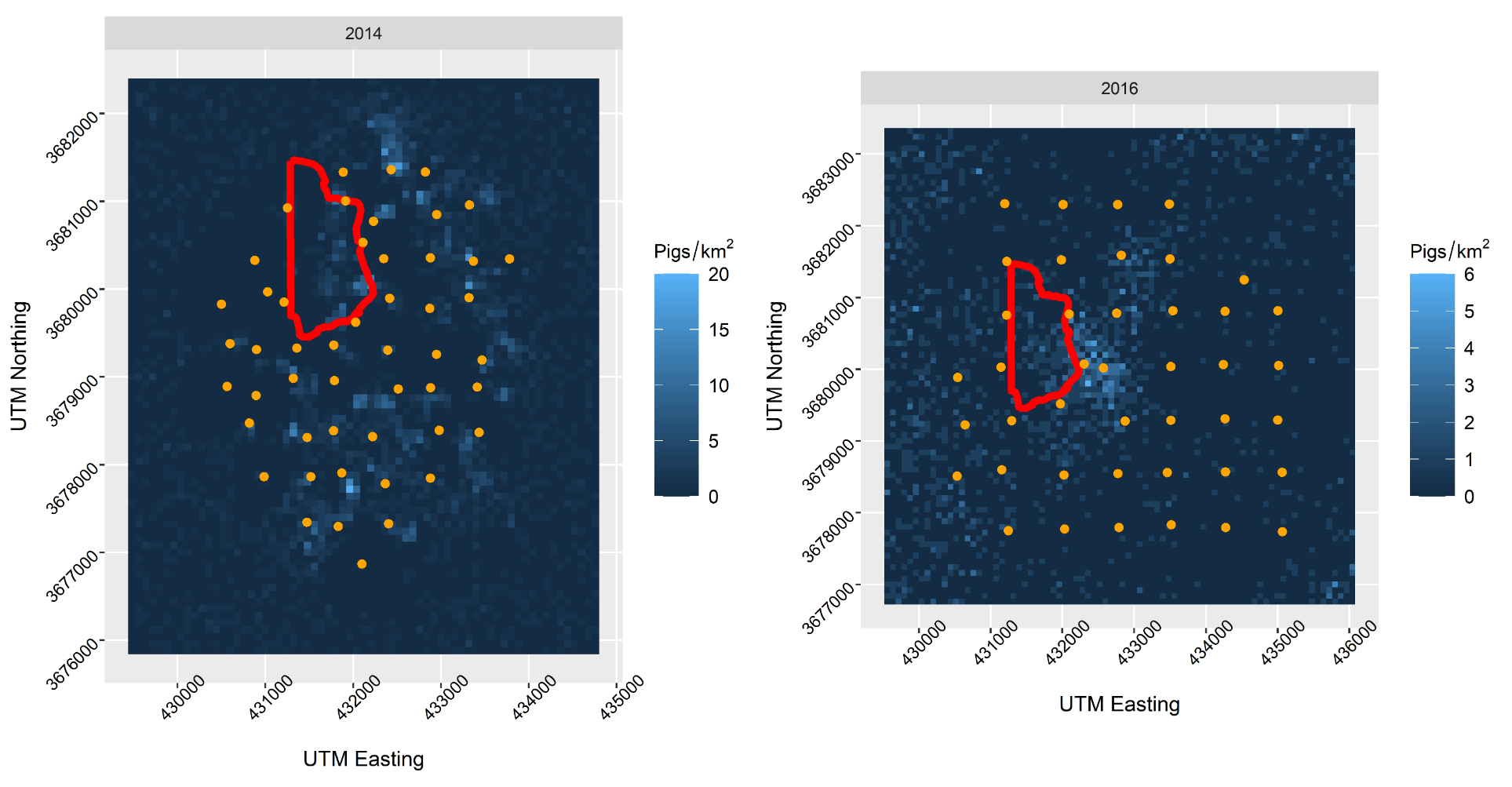


Figure S2. Mean and standard error of weekly (*n_weeks_* = 26, *n_pigs_* = 17), monthly (*n_months_* = 6, *n_pigs_* = 18), and season-long (*n_season_* = 2, *n_pigs_* = 14) two-dimensional utilization distribution (UD) overlap (Proportion UD overlap) of UDs for wild pigs (*Sus scrofa*) tracked on Savannah River Site, South Carolina, USA, in 2014 (a.i and a.ii for weekly and monthly time periods, respectively) and 2016 (b.i and b.ii for weekly and monthly time periods, respectively). Figure created in the R statistical environment version 4.0.5 (www.cran.r-project.org)^7^.


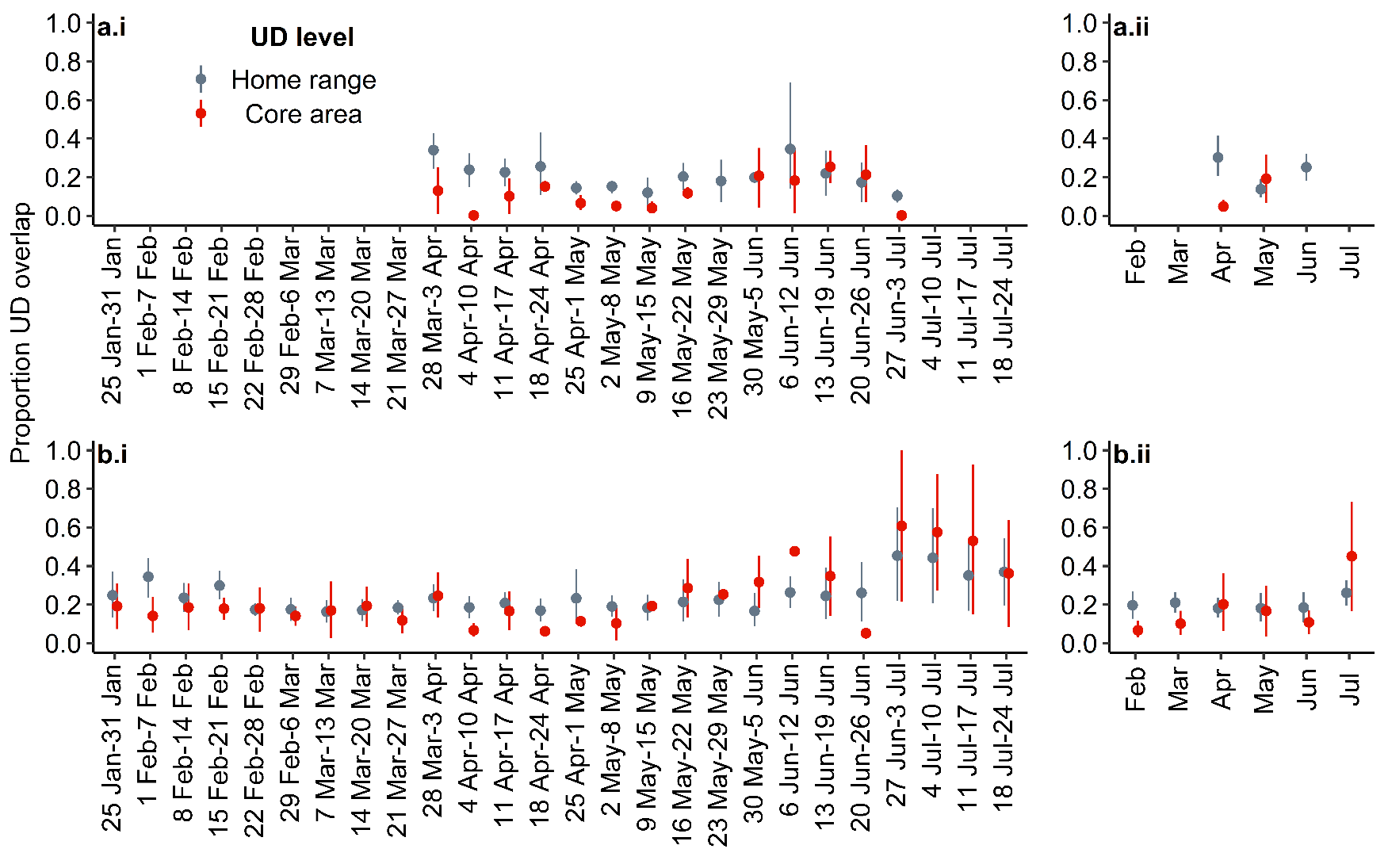


Figure S3. Mean and standard error of weekly (*n_weeks_* = 26, *n_pigs_* = 17), monthly (*n_months_* = 6, *n_pigs_* = 18), and season-long (*n_season_* = 2, *n_pigs_* = 14) three-dimensional UD overlap (Volume of intersection) of UDs for wild pigs (*Sus scrofa*) tracked on Savannah River Site, South Carolina, USA, in 2014 (a.i and a.ii for weekly and monthly time periods, respectively) and 2016 (b.i and b.ii for weekly and monthly time periods, respectively). Figure created in the R statistical environment version 4.0.5 (www.cran.r-project.org)^7^.


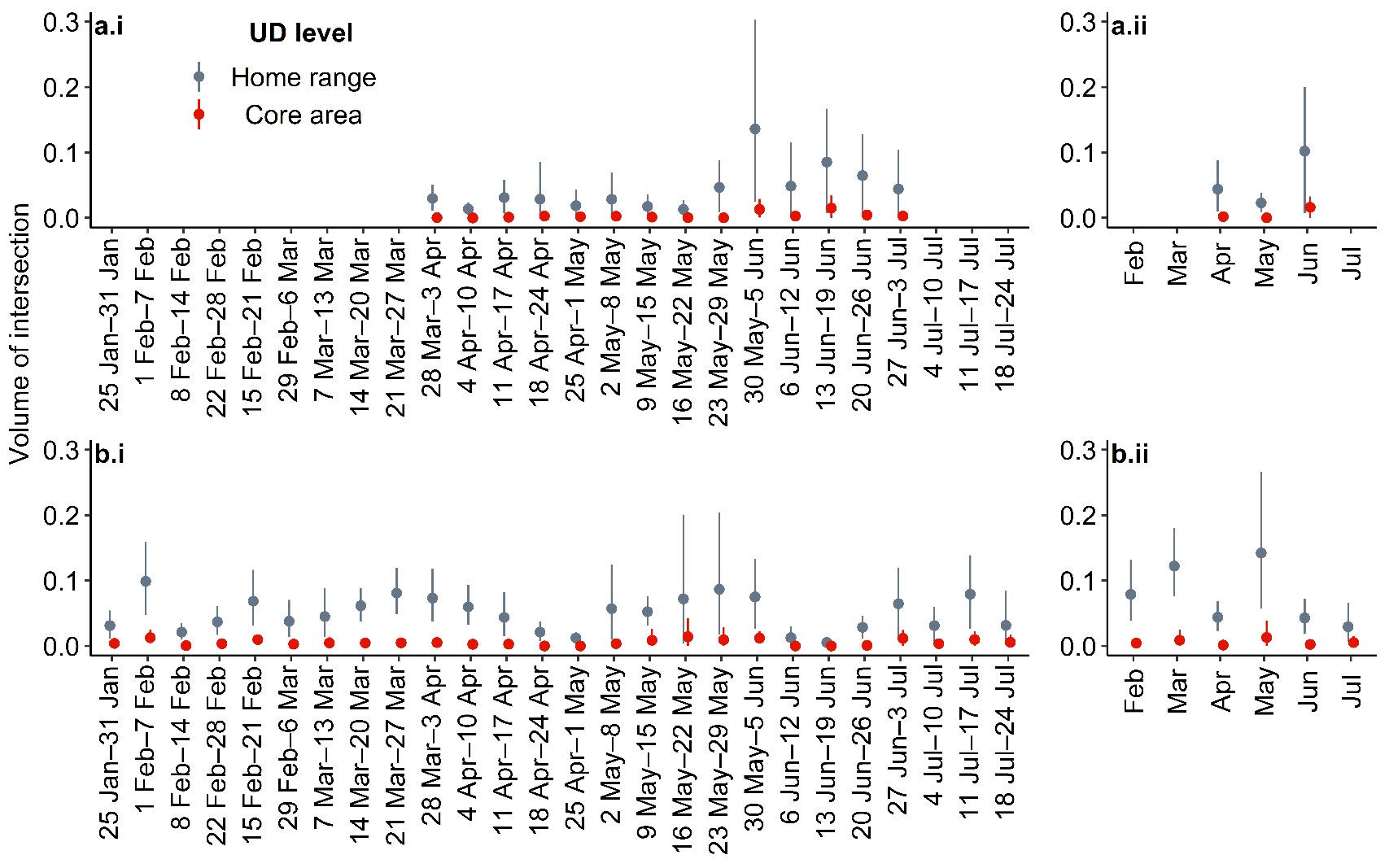


Figure S4. Predicted effects of distance from utilization distribution (UD) boundaries to the waste cells within the landfill (Distance to waste cells [km]) on UD size (ha), proportional area of overlap (2D overlap), and volume of intersection (VI) of monthly (a.i, b.i, and c.i; *n_months_* = 6, *n_pigs_* = 18), and weekly (a.ii, b.ii, and c.ii; *n_weeks_* = 26, *n_pigs_* = 17) UDs for wild pigs (*Sus scrofa*) on Savannah River Site, South Carolina, USA, in 2014 and 2016. Figure created in the R statistical environment version 4.0.5 (www.cran.r-project.org)^7^.


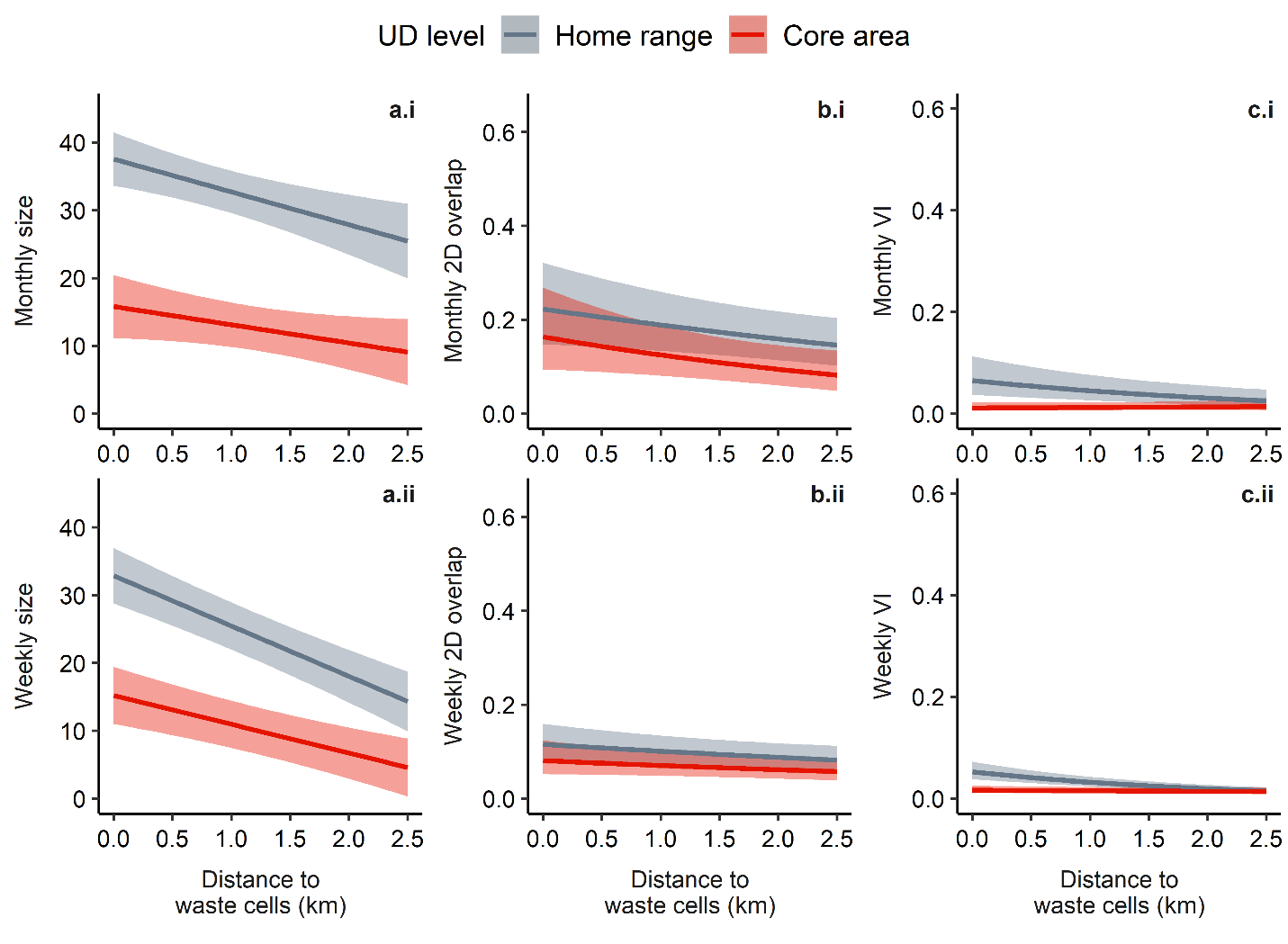


Figure S5. Predicted effects of percentage of utilization distributions (UD) that included the landfill (% UD in landfill) on UD size (ha), proportional area of overlap (2D overlap), and volume of intersection (VI) of monthly (a.i, b.i, and c.i; *n_months_* = 6, *n_pigs_* = 18), and weekly (a.ii, b.ii, and c.ii; *n_weeks_* = 26, *n_pigs_* = 17) UDs for wild pigs (*Sus scrofa*) on Savannah River Site, South Carolina, USA, in 2014 and 2016. Figure created in the R statistical environment version 4.0.5 (www.cran.r-project.org)^7^.


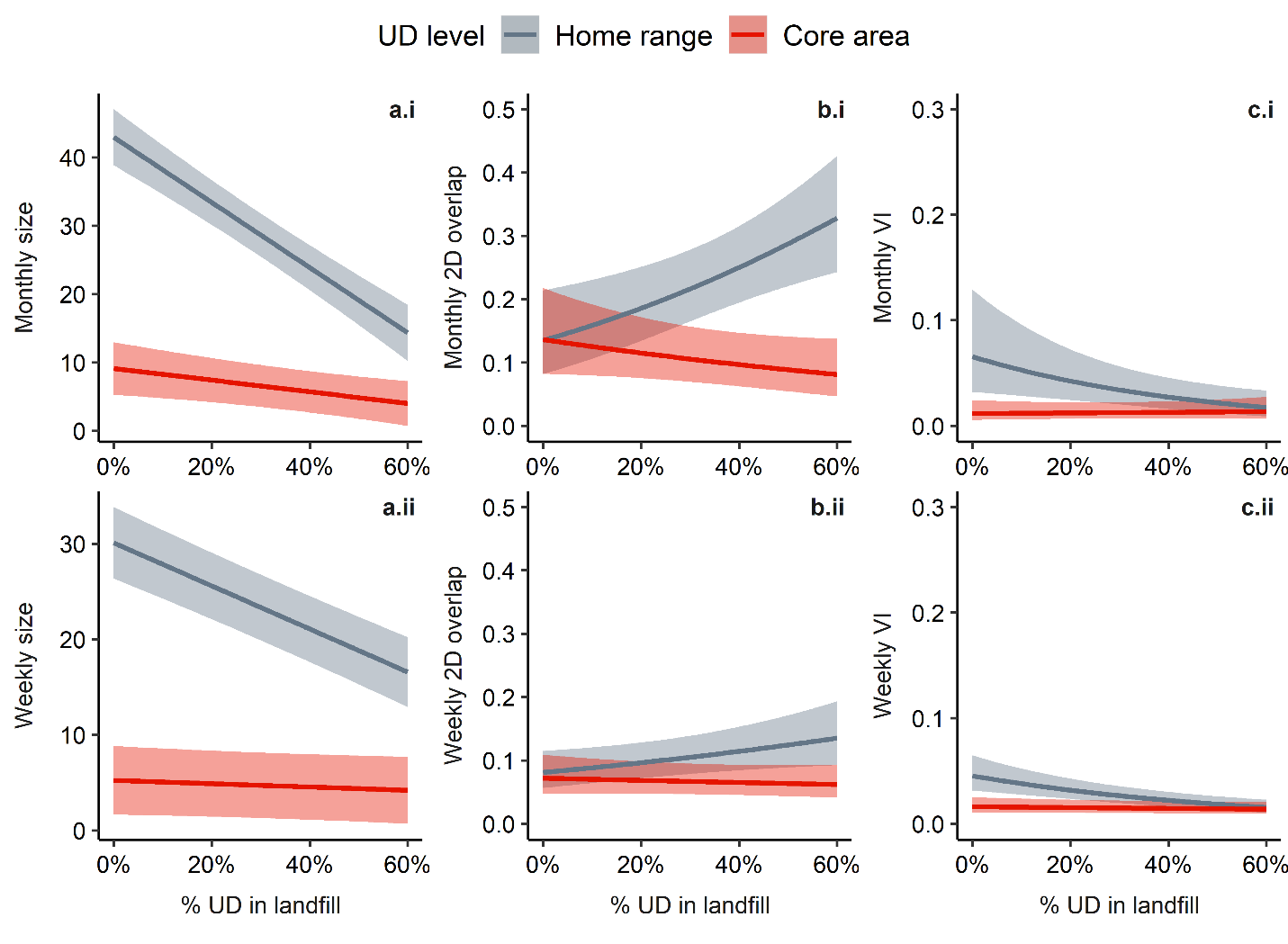


Figure S6. Season-long simultaneous locations that were 50 meters apart overlaid on core area boundaries of wild pig (*Sus scrofa*) dyads tracked in 2014 (total simultaneous dyad locations = 1,030) and 2016 (total simultaneous dyad locations = 1,294) on the Savannah River Site, South Carolina, USA. Map created using ArcGIS Pro version 2.8.1 (www.esri.com)^18^.


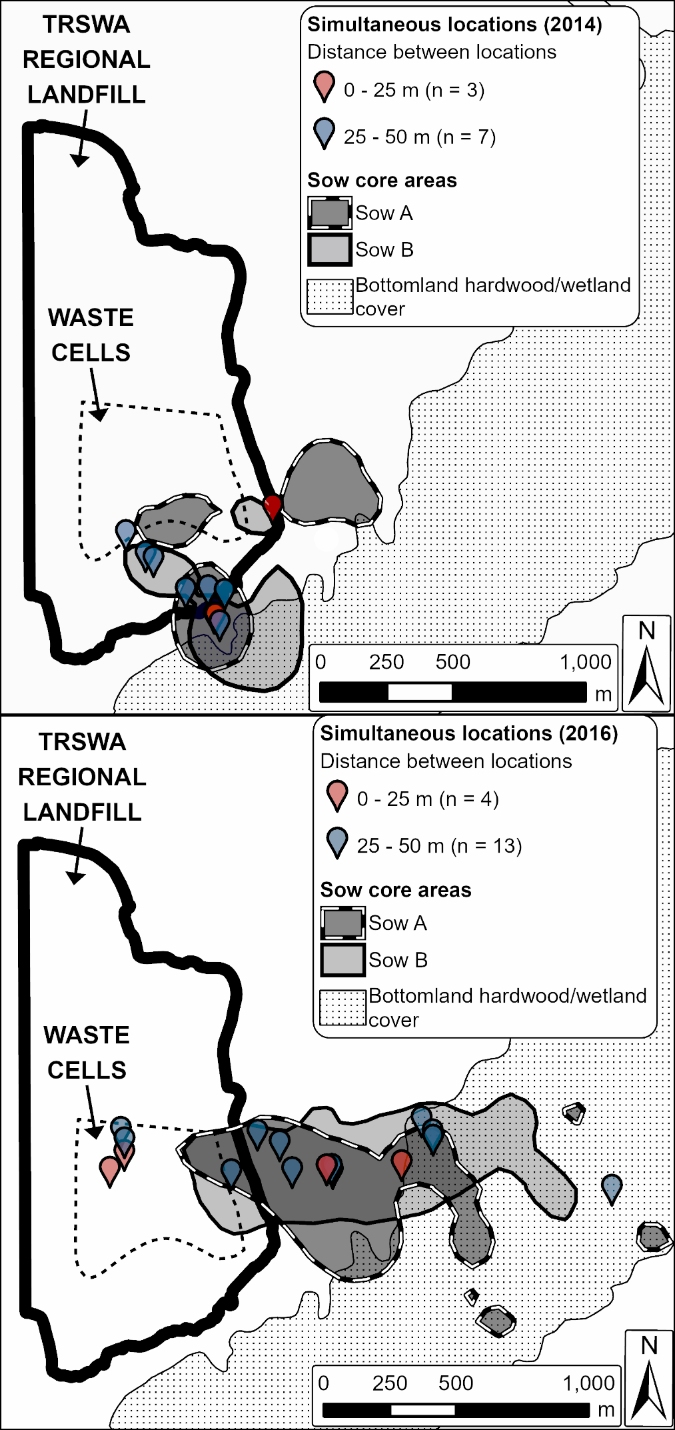


Figure S7. Mean and standard error of weekly, monthly, and season-long frequency of locations that were within 10 minutes and 50 meters apart (a.i and a.ii for weekly and monthly time periods, respectively) and half-weight association (HAI) indices calculated during periods of simultaneous use within the landfill footprint (b.i and b.ii for weekly and monthly time periods, respectively) and within the waste cells (c.i and c.ii for weekly and monthly time periods, respectively) for wild pigs (*Sus scrofa*; *n* = 11) tracked on Savannah River Site, South Carolina, USA, in 2016. Figure created in the R statistical environment version 4.0.5 (www.cran.r-project.org)^7^.


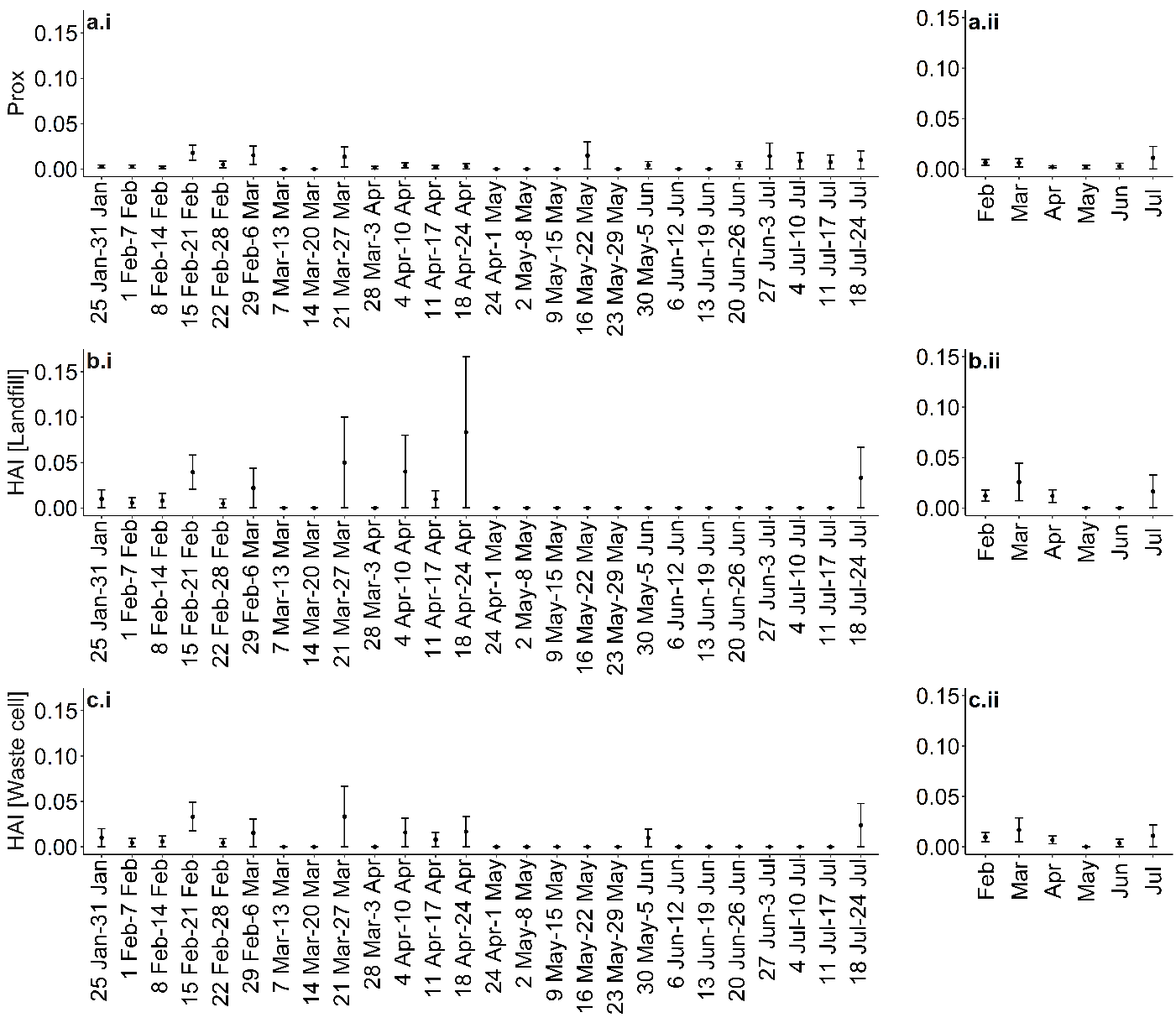


Table S1. Mean and standard deviation (SD) of wild pig (*Sus scrofa*) sounder size, body mass, temperature, barometric pressure, hectare of utilization distributions (UD) within the landfill boundary, percent of UDs within the landfill, ha of UDs within bottomland hardwoods, percent of UDs within bottomland hardwoods/wetlands, and distance from UD boundaries to the waste cells within the landfill across time periods (Season-long [*n_season_* = 2; *n_pigs_* = 14], Month [*n_months_* = 6; *n_pigs_* = 18], and Week [*n_weeks_* = 26; *n_pigs_* = 17]) and UD levels representing home ranges (95% UD contour; HR) and core areas (50% UD contour; CA) for pigs tracked on the Savannah River Site, South Carolina, USA in 2014 and 2016.

| Variable^a^ | UD level | Season-long | Month | Week |
| --- | --- | --- | --- | --- |
|  |  | Mean ± SD | Mean ± SD | Mean ± SD |
| Sounder |  | 7.5 ± 5.6 | 7.3 ± 5.9 | 7.5 ± 5.9 |
| Mass |  | 76.5 ± 18.6 | 75.9 ± 20.1 | 76.4 ± 20.5 |
| Temp |  | 22.8 ± 0.6 | 19.3 ± 5.7 | 19.0 ± 6.2 |
| Press |  | 1005.6 ± 0.3 | 1005.9 ± 1.1 | 1005.9 ± 2.6 |
| HA.UD.LF | HR | 52.5 ± 39.0 | 43.2 ± 36.9 | 37.5 ± 32.9 |
|  | CA | 7.5 ± 8.8 | 7.9 ± 9.2 | 7.4 ± 8.4 |
| %UD.LF | HR | 25.1 ± 18.6 | 22.4 ± 19.2 | 24.8 ± 21.9 |
|  | CA | 21.8 ± 23.8 | 22.8 ± 24.9 | 27.8 ± 28.0 |
| HA.UD.BLHW | HR | 55.1 ± 60.3 | 62.5 ± 57.8 | 47.5 ± 52.9 |
|  | CA | 7.3 ± 8.1 | 7.4 ± 10.4 | 6.2 ± 9.4 |
| %UD.BLHW | HR | 21.4 ± 19.6 | 25.7 ± 19.5 | 25.6 ± 22.2 |
|  | CA | 18.5 ± 19.2 | 20.2 ± 24.1 | 17.9 ± 22.2 |
| Dist.WC | HR | 0.53 ± 0.64 | 0.44 ± 0.52 | 0.41 ± 0.50 |
|  | CA | 0.81 ± 0.85 | 0.5 ± 0.51 | 0.46 ± 0.51 |

^a^ Sounder = Sounder size; Mass = body mass (kg); Temp = temperature (C°); Press = barometric pressure (mb); HA.UD.LF = hectare (ha) of utilization distributions (UD) within the landfill boundary; %UD.LF = percent of UDs within the landfill; HA.UD.BLHW = ha of UDs within bottomland hardwood/wetland cover; %UD.BLHW = percent of UDs within bottomland hardwoods/wetland cover; and Dist.WC = distance from UD boundaries to the waste cells within the landfill (km).

Table S2. Summary of candidate models for wild pig (*Sus scrofa*; *n*=14) utilization distributions (UD) size during season-long time periods (27 Mar-12 Jul) tracked on Savannah River Site, South Carolina, USA during 2014 and 2016.

| Season-long size models | ΔAIC*_c_* | AIC*_c_* weight | Model formula |
| --- | --- | --- | --- |
| UD*Landfill+Sow+BLHW | 0 | 0.76 | ~UD.lev*%UD.LF+Dist.WC+Age+Mass+Sounder+%UD.BLHW |
| Sow | 4.8 | 0.07 | ~UD.lev+Age+Mass+Sounder |
| Landfill+Sow+BLHW | 5.21 | 0.06 | ~UD.lev+Dist.WC+%UD.LF+Age+Mass+Sounder+%UD.BLHW |
| Sow+BLHW | 6.04 | 0.04 | ~UD.lev+Age+Mass+Sounder+%UD.BLHW |
| Landfill | 6.57 | 0.03 | ~UD.lev+Dist.WC+%UD.LF |
| Landfill+Sow | 7.23 | 0.02 | ~UD.lev+Dist.WC+%UD.LF+Age+Mass+Sounder |
| BLHW | 7.48 | 0.02 | ~UD.lev+%UD.BLHW |
| Landfill+BLHW | 8.4 | 0.01 | ~UD.lev+%UD.LF+%UD.BLHW |
| Null | 36.05 | 0 | ~1 |

^a^ UD.lev = utilization distribution (UD) contour level (i.e., 95% and 50% contours representing home ranges and core areas, respectively); Sounder = Sounder size; Mass = body mass (kg); Temp = temperature (C°); Press = barometric pressure (mb); HA.UD.LF = hectare (ha) of utilization distributions (UD) within the landfill boundary; %UD.LF = percent of UDs within the landfill; HA.UD.BLHW = ha of UDs within bottomland hardwood/wetland cover; %UD.BLHW = percent of UDs within bottomland hardwoods/wetland cover; and Dist.WC = distance from UD boundaries to the waste cells within the landfill (km).

Table S3. Parameter estimates and 90% confidence intervals (CI) for the top supported model of utilization distribution size (ha) for wild pigs (*Sus scrofa*) across time periods (Season-long [*n_season_* = 2; *n_pigs_* = 14], Month [*n_months_* = 6; *n_pigs_* = 18], and Week [*n_weeks_* = 26; *n_pigs_* = 17]) tracked on Savannah River Site, South Carolina, USA during 2014 and 2016. Colons separating predictors denote interaction terms and asterisks denote 90% CI of parameter estimates that did not overlap 0.

| Predictors^a^ | Season-long | Month | Week |
| --- | --- | --- | --- |
|  | β (90% CI) | β (90% CI) | β (90% CI) |
| Year |  | -3.4^*^ (-6.3 – -0.5) | -1.4 (-4.8 – 1.9) |
| UD.lev | 28.5^*^ (22.4 – 34.7) | 50.3^*^ (42.9 – 57.7) | 38.5^*^ (34.2 – 42.8) |
| %UD.LF | -6.6 (-20.4 – 7.2) | -8.5^*^ (-14.6 – -2.5) | -1.7 (-4.8 – 1.3) |
| Dist.WC | -2.0 (-4.6 – 0.6) | -7.5 (-15.5 – 0.4) | -7.1^*^ (-11.9 – -2.2) |
| Age | -8.7^*^ (-14.3 – -3.1) | -3.5^*^ (-6.8 – -0.3) | -2.2 (-6.1 – 1.7) |
| Mass | -1.5^*^ (-2.7 – -0.3) | -0.11 (-0.93 – 0.71) | -0.3 (-1.1 – 0.5) |
| Sounder | 0.7^*^ (0.2 – 1.2) | -0.12 (-0.58 – 0.34) | -0.18 (-0.62 – 0.26) |
| %UD.BLHW | -20.9^*^ (-36.3 – -5.4) | -0.7 (-6.2 – 4.9) | 0.2 (-2.5 – 2.8) |
| UD.lev : %UD.LF | -27.9^*^ (-47.7 – -8.1) | -39.2^*^ (-48.6 – -29.7) | -20.8^*^ (-25.4 – -16.2) |
| UD.lev : Dist.WC |  | -1.9 (-9.8 – 6.0) | -5.2^*^ (-9.5 – -0.8) |
| UD.lev : Age |  | -5.8^*^ (-9.4 – -2.2) | -7.0^*^ (-9.1 – -4.9) |
| UD.lev : Mass |  | -1.7^*^ (-2.6 – -0.8) | -1.5^*^ (-2.0 – -1.0) |
| UD.lev : Sounder |  | 0.6^*^ (0.02 – 1.2) | 0.51^*^ (0.22 – 0.80) |
| UD.lev : %UD.BLHW |  | -23.9^*^ (-33.6 – -14.3) | -16.6^*^ (-21.0 – -12.2) |
| Dist.WC : %UD.LF |  | -1.1 (-6.9 – 4.9) | -4.3^*^ (-7.5 – -1.1) |
| Dist.WC : Age |  | 1.6 (-3.1 – 6.2) | 2.3 (-0.9 – 5.4) |
| Dist.WC : Mass |  | 0.6 (-0.2 – 1.5) | 0.43 (-0.16 – 1.02) |
| Dist.WC : Sounder |  | -0.4 (-1.1 – 0.2) | -0.43^*^ (-0.84 – -0.02) |
| Dist.WC : %UD.BLHW |  | 1.3 (-6.5 – 9.0) | 1.7 (-1.6 – 4.9) |
| UD.lev : Dist.WC : %UD.LF |  | 18.4^*^ (7.8 – 28.9) | 7.2^*^ (2.3 – 12.1) |
| UD.lev : Dist.WC : Age |  | -4.0 (-9.1 – 1.0) | 0.2 (-2.7 – 3.0) |
| UD.lev : Dist.WC : Mass |  | -0.2 (-1.2 – 0.7) | 0.33 (-0.17 – 0.84) |
| UD.lev : Dist.WC : Sounder |  | 0.19 (-0.60 – 0.98) | -0.08 (-0.47 – 0.31) |
| UD.lev : Dist.WC : %UD.BLHW |  | -8.5 (-20.1 – 3.2) | -7.8^*^ (-13.1 – -2.5) |

^a^ UD.lev = utilization distribution (UD) contour level (i.e., 95% and 50% contours representing home ranges and core areas, respectively); Sounder = Sounder size; Mass = body mass (kg); Temp = temperature (C°); Press = barometric pressure (mb); HA.UD.LF = hectare (ha) of utilization distributions (UD) within the landfill boundary; %UD.LF = percent of UDs within the landfill; HA.UD.BLHW = ha of UDs within bottomland hardwood/wetland cover; %UD.BLHW = percent of UDs within bottomland hardwoods/wetland cover; and Dist.WC = distance from UD boundaries to the waste cells within the landfill (km).

Table S4. Summary of candidate models for size of monthly utilization distributions (UD) of wild pigs (*Sus scrofa*; n=18) tracked on Savannah River Site, South Carolina, USA during 2014 and 2016.

| Candidate models | ΔAIC*_c_* | AIC*_c_* weight | Model formula |
| --- | --- | --- | --- |
| Landfill*Sow+BLHW | 0 | 0.99 | ~Year+UD.lev*Dist.WC*(%UD.LF+Age+Mass+Sounder)+%UD.BLHW |
| Landfill*Sow+BLHW2 | 9.98 | 0.01 | ~Year+UD.lev*(Dist.WC+%UD.LF+Age+Mass+Sounder)+%UD.BLHW |
| Landfill*Sow | 10.49 | 0.01 | ~Year+UD.lev*Dist.WC*(%UD.LF+Age+Mass+Sounder) |
| Landfill*BLHW | 17.09 | 0 | ~Year+UD.lev*Dist.WC*(%UD.LF+%UD.BLHW) |
| Landfill.int | 32.86 | 0 | ~Year+UD.lev*Dist.WC*%UD.LF |
| Landfill+Sow+BLHW | 42.49 | 0 | ~Year+UD.lev+Dist.WC+%UD.LF+Age+Mass+Sounder+%UD.BLHW |
| Landfill+Sow | 42.96 | 0 | ~Year+UD.lev+Dist.WC+%UD.LF+Age+Mass+Sounder |
| Landfill+Sow+BLHW+Weather | 46.11 | 0 | ~Year+UD.lev+Dist.WC+%UD.LF+Age+Mass+Sounder+%UD.BLHW+Temp+Press |
| Landfill+Sow+Weather | 46.57 | 0 | ~Year+UD.lev+Dist.WC+%UD.LF+Age+Mass+Sounder+%UD.BLHW |
| Landfill+BLHW | 47.09 | 0 | ~Year+UD.lev+Dist.WC+%UD.LF+%UD.BLHW |
| Landfill | 47.11 | 0 | ~Year+UD.lev+Dist.WC+%UD.LF |
| Sow.int | 48.93 | 0 | ~Year+UD.lev*(Age+Mass+Sounder) |
| Landfill+BLHW+Weather | 50.62 | 0 | ~Year+UD.lev+Dist.WC+%UD.LF+%UD.BLHW+Temp+Press |
| Sow | 60.36 | 0 | ~Year+UD.lev+Age+Mass+Sounder |
| Sow+BLHW | 62.32 | 0 | ~Year+UD.lev+Age+Mass+Sounder+%UD.BLHW |
| Sow+Weather | 64.24 | 0 | ~Year+UD.lev+Age+Mass+Sounder+Temp+Press |
| BLHW | 66.08 | 0 | ~Year+UD.lev+%UD.BLHW |
| Sow+BLHW+Weather | 66.2 | 0 | ~Year+UD.lev+Age+Mass+Sounder+%UD.BLHW+Temp+Press |
| BLHW.int | 67.51 | 0 | ~Year+UD.lev*%UD.BLHW |
| Weather | 68.11 | 0 | ~Year+UD.lev+Temp+Press |
| Null | 209.22 | 0 | ~1 |

^a^ UD.lev = utilization distribution (UD) contour level (i.e., 95% and 50% contours representing home ranges and core areas, respectively); Sounder = Sounder size; Mass = body mass (kg); Temp = temperature (C°); Press = barometric pressure (mb); HA.UD.LF = hectare (ha) of utilization distributions (UD) within the landfill boundary; %UD.LF = percent of UDs within the landfill; HA.UD.BLHW = ha of UDs within bottomland hardwood/wetland cover; %UD.BLHW = percent of UDs within bottomland hardwoods/wetland cover; and Dist.WC = distance from UD boundaries to the waste cells within the landfill (km).

Table S5. Summary of candidate models for size of weekly utilization distributions (UD) of wild pigs (*Sus scrofa*; n=17) tracked on Savannah River Site, South Carolina, USA during 2014 and 2016.

| Weekly size models | ΔAIC*_c_* | AIC*_c_* weight | Model formula |
| --- | --- | --- | --- |
| Landfill*Sow+BLHW | 0 | 1 | ~Year+UD.lev*Dist.WC*(%UD.LF+Age+Mass+Sounder)+%UD.BLHW |
| Landfill*Sow+BLHW2 | 21.53 | 0 | ~Year+UD.lev*(Dist.WC+%UD.LF+Age+Mass+Sounder)+%UD.BLHW |
| Landfill*Sow | 35.77 | 0 | ~Year+UD.lev*Dist.WC*(%UD.LF+Age+Mass+Sounder) |
| Landfill*BLHW | 48.09 | 0 | ~Year+UD.lev*Dist.WC*(%UD.LF+%UD.BLHW) |
| Landfill.int | 91.4 | 0 | ~Year+UD.lev*Dist.WC*%UD.LF |
| Landfill+Sow+BLHW+Weather | 105 | 0 | ~Year+UD.lev+Dist.WC+%UD.LF+Age+Mass+Sounder+%UD.BLHW+Temp+Press |
| Landfill+BLHW+Weather | 107.1 | 0 | ~Year+UD.lev+Dist.WC+%UD.LF+%UD.BLHW+Temp+Press |
| Landfill+Sow+BLHW | 107.81 | 0 | ~Year+UD.lev+Dist.WC+%UD.LF+Age+Mass+Sounder+%UD.BLHW |
| Landfill+Sow+Weather | 107.85 | 0 | ~Year+UD.lev+Dist.WC+%UD.LF+Age+Mass+Sounder+Temp+Press |
| Landfill+Sow | 110.31 | 0 | ~Year+UD.lev+Dist.WC+%UD.LF+Age+Mass+Sounder |
| Landfill+BLHW | 110.55 | 0 | ~Year+UD.lev+Dist.WC+%UD.LF+%UD.BLHW |
| Landfill | 113 | 0 | ~Year+UD.lev+Dist.WC+%UD.LF |
| Sow.int | 156.4 | 0 | ~Year+UD.lev*(Age+Mass+Sounder) |
| Sow+Weather | 187.78 | 0 | ~Year+UD.lev+Age+Mass+Sounder+Temp+Press |
| Sow | 187.79 | 0 | ~Year+UD.lev+Age+Mass+Sounder |
| Sow+BLHW+Weather | 188.41 | 0 | ~Year+UD.lev+Age+Mass+Sounder+%UD.BLHW+Temp+Press |
| Sow+BLHW | 188.56 | 0 | ~Year+UD.lev+Age+Mass+Sounder+%UD.BLHW |
| Weather | 190.22 | 0 | ~Year+UD.lev+Temp+Press |
| BLHW | 191.1 | 0 | ~Year+UD.lev+%UD.BLHW |
| BLHW.int | 192.55 | 0 | ~Year+UD.lev*%UD.BLHW |
| Null | 596.18 | 0 | ~1 |

^a^ UD.lev = utilization distribution (UD) contour level (i.e., 95% and 50% contours representing home ranges and core areas, respectively); Sounder = Sounder size; Mass = body mass (kg); Temp = temperature (C°); Press = barometric pressure (mb); HA.UD.LF = hectare (ha) of utilization distributions (UD) within the landfill boundary; %UD.LF = percent of UDs within the landfill; HA.UD.BLHW = ha of UDs within bottomland hardwood/wetland cover; %UD.BLHW = percent of UDs within bottomland hardwoods/wetland cover; and Dist.WC = distance from UD boundaries to the waste cells within the landfill (km).

Table S6. Summary of candidate models for two-dimensional utilization distribution (UD) overlap, measured as the proportional area of UD overlap, for season-long (27 Mar-12 Jul) UDs of wild pigs (*Sus scrofa*; *n*=14) tracked on Savannah River Site, South Carolina, USA during 2014 and 2016.

| Candidate models | ΔAIC*_c_* | AIC*_c_*  weight | Model formula |
| --- | --- | --- | --- |
| Landfill*Sow+BLHW | 0 | 1 | ~Year+UD.lev*Dist.WC*(%UD.LF+Age+Mass+Sounder)+%UD.BLHW |
| Landfill*Sow+BLHW2 | 15.21 | 0 | ~Year+UD.lev*(Dist.WC+%UD.LF+Age+Mass+Sounder)+%UD.BLHW |
| Landfill.int2 | 17.11 | 0 | ~Year+UD.lev*Dist.WC*%UD.LF |
| Landfill | 23.11 | 0 | ~Year+UD.lev+Dist.WC+%UD.LF |
| Landfill+Sow | 24.43 | 0 | ~Year+UD.lev+Dist.WC+%UD.LF+Age+Mass+Sounder |
| Landfill+BLHW | 24.64 | 0 | ~Year+UD.lev+Dist.WC+%UD.LF+%UD.BLHW |
| Sow | 25.47 | 0 | ~Year+UD.lev+Age+Mass+Sounder |
| Landfill+Sow+BLHW | 26.43 | 0 | ~Year+UD.lev+Dist.WC+%UD.LF+Age+Mass+Sounder+%UD.BLHW |
| Landfill.int | 26.69 | 0 | ~Year+UD.lev+Dist.WC*%UD.LF |
| Sow+BLHW | 26.97 | 0 | ~Year+UD.lev+Age+Mass+Sounder+%UD.BLHW |
| BLHW | 27.66 | 0 | ~Year+UD.lev+%UD.BLHW |
| Landfill*Sow | 28.17 | 0 | ~Year+UD.lev*Dist.WC*(%UD.LF+Age+Mass+Sounder) |
| Sow.int | 29.38 | 0 | ~Year+UD.lev*(Age+Mass+Sounder) |
| BLHW.int | 29.64 | 0 | ~Year+UD.lev+%UD.BLHW |
| Sow*BLHW | 31.63 | 0 | ~Year+UD.lev*(Age+Mass+Sounder+%UD.BLHW) |
| Null | 32.45 | 0 | ~1 |

^a^ UD.lev = utilization distribution (UD) contour level (i.e., 95% and 50% contours representing home ranges and core areas, respectively); Sounder = Sounder size; Mass = body mass (kg); Temp = temperature (C°); Press = barometric pressure (mb); HA.UD.LF = hectare (ha) of utilization distributions (UD) within the landfill boundary; %UD.LF = percent of UDs within the landfill; HA.UD.BLHW = ha of UDs within bottomland hardwood/wetland cover; %UD.BLHW = percent of UDs within bottomland hardwoods/wetland cover; and Dist.WC = distance from UD boundaries to the waste cells within the landfill (km).

Table S7. Summary of candidate models for two-dimensional utilization distribution (UD) overlap, measured as the proportional area of UD overlap, for monthly UDs of wild pigs (*Sus scrofa*; *n*=18) tracked on Savannah River Site, South Carolina, USA during 2014 and 2016.

| Candidate models | ΔAIC*_c_* | AIC*_c_*  weight | Model formula |
| --- | --- | --- | --- |
| Landfill*Sow+BLHW | 0 | 0.76 | ~Year+UD.lev*Dist.WC*(%UD.LF+Age+Mass+Sounder)+%UD.BLHW |
| Landfill.int | 4.17 | 0.09 | ~Year+UD.lev+Dist.WC*%UD.LF |
| Landfill | 4.95 | 0.03 | ~Year+UD.lev+Dist.WC+%UD.LF |
| Landfill+BLHW | 6.35 | 0.02 | ~Year+UD.lev+Dist.WC+%UD.LF+%UD.BLHW |
| Weather | 7.62 | 0.01 | ~Year+UD.lev+Temp+Press |
| BLHW | 7.87 | 0.01 | ~Year+UD.lev+%UD.BLHW |
| Landfill+Sow | 8.25 | 0.01 | ~Year+UD.lev+Dist.WC+%UD.LF+Age+Mass+Sounder |
| Sow | 8.78 | 0.01 | ~Year+UD.lev+Age+Mass+Sounder |
| Landfill*BLHW | 9.06 | 0.01 | ~Year+UD.lev*(Dist.WC+%UD.LF+%UD.BLHW) |
| Landfill+BLHW+Weather | 9.16 | 0.01 | ~Year+UD.lev+Dist.WC+%UD.LF+%UD.BLHW+Temp+Press |
| BLHW.int | 9.51 | 0 | ~Year+UD.lev*%UD.BLHW |
| Sow+Weather | 10.56 | 0 | ~Year+UD.lev+Age+Mass+Sounder+Temp+Press |
| Landfill+Sow+BLHW | 10.63 | 0 | ~Year+UD.lev+Dist.WC+%UD.LF+Age+Mass+Sounder+%UD.BLHW |
| Sow+BLHW | 11.05 | 0 | ~Year+UD.lev+Age+Mass+Sounder+%UD.BLHW |
| Landfill+Sow+Weather | 11.15 | 0 | ~Year+UD.lev+Dist.WC+%UD.LF+Age+Mass+Sounder+Temp+Press |
| Landfill*Sow+BLHW2 | 11.42 | 0 | ~Year+UD.lev*(Dist.WC+%UD.LF+Age+Mass+Sounder)+%UD.BLHW |
| Sow+BLHW+Weather | 12.57 | 0 | ~Year+UD.lev+Age+Mass+Sounder+%UD.BLHW+Temp+Press |
| Sow.int | 12.98 | 0 | ~Year+UD.lev+(Age*Mass*Sounder) |
| Landfill+Sow+BLHW+Weather | 13.25 | 0 | ~Year+UD.lev+Dist.WC+%UD.LF+Age+Mass+Sounder+%UD.BLHW+Temp+Press |
| Null | 14.39 | 0 | ~1 |
| Landfill*Sow | 21.26 | 0 | ~Year+UD.lev*Dist.WC*(%UD.LF+Age+Mass+Sounder) |

^a^ UD.lev = utilization distribution (UD) contour level (i.e., 95% and 50% contours representing home ranges and core areas, respectively); Sounder = Sounder size; Mass = body mass (kg); Temp = temperature (C°); Press = barometric pressure (mb); HA.UD.LF = hectare (ha) of utilization distributions (UD) within the landfill boundary; %UD.LF = percent of UDs within the landfill; HA.UD.BLHW = ha of UDs within bottomland hardwood/wetland cover; %UD.BLHW = percent of UDs within bottomland hardwoods/wetland cover; and Dist.WC = distance from UD boundaries to the waste cells within the landfill (km).

Table S8. Summary of candidate models for two-dimensional utilization distribution (UD) overlap, measured as the proportional area of UD overlap, for weekly UDs of wild pigs (*Sus scrofa*; *n*=17) tracked on Savannah River Site, South Carolina, USA during 2014 and 2016.

| Candidate models | ΔAIC*_c_* | AIC*_c_*  weight | Model formula |
| --- | --- | --- | --- |
| Landfill*Sow+BLHW | 0 | 0.42 | ~Year+UD.lev*Dist.WC*(%UD.LF+Age+Mass+Sounder)+%UD.BLHW |
| Sow+BLHW+Weather | 1.75 | 0.17 | ~Year+UD.lev+Age+Mass+Sounder+%UD.BLHW+Temp+Press |
| Landfill+Sow+BLHW+Weather | 2.03 | 0.15 | ~Year+UD.lev+Dist.WC+%UD.LF+Age+Mass+Sounder+%UD.BLHW+Temp+Press |
| Landfill+Sow+Weather | 2.09 | 0.15 | ~Year+UD.lev+Dist.WC+%UD.LF+Age+Mass+Sounder+Temp+Press |
| Sow+Weather | 3.25 | 0.08 | ~Year+UD.lev+Age+Mass+Sounder+Temp+Press |
| Landfill*BLHW | 7.8 | 0.01 | ~Year+UD.lev*(Dist.WC+%UD.LF+%UD.BLHW) |
| Weather | 9.9 | 0 | ~Year+UD.lev+Temp+Press |
| Landfill+BLHW+Weather | 10.34 | 0 | ~Year+UD.lev+Dist.WC+%UD.LF+%UD.BLHW+Temp+Press |
| BLHW.int | 11.48 | 0 | ~Year+UD.lev*%UD.BLHW |
| Landfill.int | 11.52 | 0 | ~Year+UD.lev+Dist.WC*%UD.LF |
| Landfill*Sow | 13.29 | 0 | ~Year+UD.lev*Dist.WC*(%UD.LF+Age+Mass+Sounder) |
| Landfill+Sow+BLHW | 14.87 | 0 | ~Year+UD.lev+Dist.WC+%UD.LF+Age+Mass+Sounder+%UD.BLHW |
| Landfill+Sow | 14.89 | 0 | ~Year+UD.lev+Dist.WC+%UD.LF+Age+Mass+Sounder |
| Sow+BLHW | 15.46 | 0 | ~Year+UD.lev+Age+Mass+Sounder+%UD.BLHW |
| Sow | 16.3 | 0 | ~Year+UD.lev+Age+Mass+Sounder |
| Landfill*Sow+BLHW2 | 19.07 | 0 | ~Year+UD.lev*(Dist.WC+%UD.LF+Age+Mass+Sounder)+%UD.BLHW |
| BLHW | 20.04 | 0 | ~Year+UD.lev+%UD.BLHW |
| Landfill | 20.23 | 0 | ~Year+UD.lev+Dist.WC+%UD.LF |
| Landfill+BLHW | 20.53 | 0 | ~Year+UD.lev+Dist.WC+%UD.LF+%UD.BLHW |
| Sow.int | 21.66 | 0 | ~Year+UD.lev*(Age+Mass+Sounder) |
| Null | 48.43 | 0 | ~1 |

^a^ UD.lev = utilization distribution (UD) contour level (i.e., 95% and 50% contours representing home ranges and core areas, respectively); Sounder = Sounder size; Mass = body mass (kg); Temp = temperature (C°); Press = barometric pressure (mb); HA.UD.LF = hectare (ha) of utilization distributions (UD) within the landfill boundary; %UD.LF = percent of UDs within the landfill; HA.UD.BLHW = ha of UDs within bottomland hardwood/wetland cover; %UD.BLHW = percent of UDs within bottomland hardwoods/wetland cover; and Dist.WC = distance from UD boundaries to the waste cells within the landfill (km).

Table S9. Summary of candidate models for three-dimensional utilization distribution (UD) overlap, measured as the volume of intersection, of season-long (27 Mar-12 Jul) UDs of wild pigs (*Sus scrofa*; *n*=14) tracked on Savannah River Site, South Carolina, USA during 2014 and 2016.

| Candidate models | ΔAIC*_c_* | AIC*_c_*  weight | Model formula |
| --- | --- | --- | --- |
| Landfill*Sow+BLHW | 0 | 0.94 | ~Year+UD.lev*Dist.WC*(%UD.LF+Age+Mass+Sounder)+%UD.BLHW |
| Landfill*Sow+BLHW2 | 5.45 | 0.04 | ~Year+UD.lev*(Dist.WC+%UD.LF+Age+Mass+Sounder)+%UD.BLHW |
| Landfill+Sow+BLHW | 5.96 | 0.03 | ~Year+UD.lev+Dist.WC+%UD.LF+Age+Mass+Sounder+%UD.BLHW |
| Sow*BLHW | 8.14 | 0.01 | ~Year+UD.lev*(Age+Mass+Sounder+%UD.BLHW) |
| Sow+BLHW | 13.8 | 0 | ~Year+UD.lev+Age+Mass+Sounder+%UD.BLHW |
| BLHW | 17.91 | 0 | ~Year+UD.lev+%UD.BLHW |
| Sow | 18.9 | 0 | ~Year+UD.lev+Age+Mass+Sounder |
| BLHW.int | 18.97 | 0 | ~Year+UD.lev*%UD.BLHW |
| Landfill | 20.43 | 0 | ~Year+UD.lev+Dist.WC+%UD.LF |
| Landfill+BLHW | 20.75 | 0 | ~Year+UD.lev+Dist.WC+%UD.LF+%UD.BLHW |
| Null | 21.4 | 0 | ~1 |
| Landfill+Sow | 21.47 | 0 | ~Year+UD.lev+Dist.WC+%UD.LF+Age+Mass+Sounder |
| Landfill.int | 22.01 | 0 | ~Year+UD.lev+Dist.WC*%UD.LF |
| Landfill*Sow | 22.69 | 0 | ~Year+UD.lev*Dist.WC*(%UD.LF+Age+Mass+Sounder) |
| Sow.int | 24.1 | 0 | ~Year+UD.lev*(Age+Mass+Sounder) |
| Landfill.int2 | 26.17 | 0 | ~Year+UD.lev*Dist.WC*%UD.LF |

^a^ UD.lev = utilization distribution (UD) contour level (i.e., 95% and 50% contours representing home ranges and core areas, respectively); Sounder = Sounder size; Mass = body mass (kg); Temp = temperature (C°); Press = barometric pressure (mb); HA.UD.LF = hectare (ha) of utilization distributions (UD) within the landfill boundary; %UD.LF = percent of UDs within the landfill; HA.UD.BLHW = ha of UDs within bottomland hardwood/wetland cover; %UD.BLHW = percent of UDs within bottomland hardwoods/wetland cover; and Dist.WC = distance from UD boundaries to the waste cells within the landfill (km).

Table S10. Summary of candidate models for three-dimensional utilization distribution (UD) overlap, measured as the volume of intersection, for monthly UDs of wild pigs (*Sus* *scrofa*; *n*=18) tracked on Savannah River Site, South Carolina, USA during 2014 and 2016.

| Candidate models | ΔAIC*_c_* | AIC*_c_*  weight | Model formula |
| --- | --- | --- | --- |
| Landfill*Sow | 0 | 0.97 | ~Year+UD.lev*Dist.WC*(%UD.LF+Age+Mass+Sounder) |
| Landfill*Sow+BLHW | 7.82 | 0.02 | ~Year+UD.lev*Dist.WC*(%UD.LF+Age+Mass+Sounder)+%UD.BLHW |
| Landfill*BLHW | 9.98 | 0.01 | ~Year+UD.lev*(Dist.WC+%UD.LF+%UD.BLHW) |
| Landfill+BLHW | 14.89 | 0 | ~Year+UD.lev+Dist.WC+%UD.LF+%UD.BLHW |
| Sow | 15.39 | 0 | ~Year+UD.lev+Age+Mass+Sounder |
| Landfill | 16.5 | 0 | ~Year+UD.lev+Dist.WC+%UD.LF |
| BLHW | 16.53 | 0 | ~Year+UD.lev+%UD.BLHW |
| Landfill+Sow | 16.84 | 0 | ~Year+UD.lev+Dist.WC+%UD.LF+Age+Mass+Sounder |
| Landfill+Sow+BLHW | 17.08 | 0 | ~Year+UD.lev+Dist.WC+%UD.LF+Age+Mass+Sounder+%UD.BLHW |
| Landfill.int | 17.59 | 0 | ~Year+UD.lev+Dist.WC*%UD.LF |
| Sow+BLHW | 17.9 | 0 | ~Year+UD.lev+Age+Mass+Sounder+%UD.BLHW |
| Weather | 18.3 | 0 | ~Year+UD.lev+Temp+Press |
| BLHW.int | 18.35 | 0 | ~Year+UD.lev*%UD.BLHW |
| Sow+Weather | 18.43 | 0 | ~Year+UD.lev+Age+Mass+Sounder+Temp+Press |
| Landfill*Sow+BLHW2 | 18.58 | 0 | ~Year+UD.lev*(Dist.WC+%UD.LF+Age+Mass+Sounder)+%UD.BLHW |
| Sow.int | 19.28 | 0 | ~Year+UD.lev*(Age+Mass+Sounder) |
| Landfill+BLHW+Weather | 19.93 | 0 | ~Year+UD.lev+Dist.WC+%UD.LF+%UD.BLHW+Temp+Press |
| Sow+BLHW+Weather | 21.01 | 0 | ~Year+UD.lev+Age+Mass+Sounder+%UD.BLHW+Temp+Press |
| Landfill+Sow+Weather | 21.21 | 0 | ~Year+UD.lev+Dist.WC+%UD.LF+Age+Mass+Sounder+Temp+Press |
| Landfill+Sow+BLHW+Weather | 22.06 | 0 | ~Year+UD.lev+Dist.WC+%UD.LF+Age+Mass+Sounder+%UD.BLHW+Temp+Press |
| Null | 49.38 | 0 | ~1 |

^a^ UD.lev = utilization distribution (UD) contour level (i.e., 95% and 50% contours representing home ranges and core areas, respectively); Sounder = Sounder size; Mass = body mass (kg); Temp = temperature (C°); Press = barometric pressure (mb); HA.UD.LF = hectare (ha) of utilization distributions (UD) within the landfill boundary; %UD.LF = percent of UDs within the landfill; HA.UD.BLHW = ha of UDs within bottomland hardwood/wetland cover; %UD.BLHW = percent of UDs within bottomland hardwoods/wetland cover; and Dist.WC = distance from UD boundaries to the waste cells within the landfill (km).

Table S11. Summary of candidate models for three-dimensional utilization distribution (UD) overlap, measured as the volume of intersection, for weekly UDs of wild pigs (*Sus scrofa*; *n*=17) tracked on Savannah River Site, South Carolina, USA during 2014 and 2016.

| Candidate models | ΔAIC*_c_* | AIC*_c_*  weight | Model formula |
| --- | --- | --- | --- |
| Landfill*Sow+BLHW | 0 | 0.6 | ~Year+UD.lev*Dist.WC*(%UD.LF+Age+Mass+Sounder)+%UD.BLHW |
| Landfill*BLHW | 1.44 | 0.29 | ~Year+UD.lev*Dist.WC*(%UD.LF+%UD.BLHW) |
| Landfill*Sow+BLHW2 | 5.77 | 0.03 | ~Year+UD.lev*(Dist.WC+%UD.LF+Age+Mass+Sounder)+%UD.BLHW |
| Landfill+Sow+BLHW | 5.95 | 0.03 | ~Year+UD.lev+Dist.WC+%UD.LF+Age+Mass+Sounder+%UD.BLHW |
| Landfill+Sow | 6.62 | 0.02 | ~Year+UD.lev+Dist.WC+%UD.LF+Age+Mass+Sounder |
| Landfill+Sow+BLHW+Weather | 9.34 | 0.01 | ~Year+UD.lev+Dist.WC+%UD.LF+Age+Mass+Sounder+%UD.BLHW+Temp+Press |
| Landfill+Sow+Weather | 10.1 | 0 | ~Year+UD.lev+Dist.WC+%UD.LF+Age+Mass+Sounder+Temp+Press |
| Landfill.int | 11.09 | 0 | ~Year+UD.lev+Dist.WC*%UD.LF |
| Landfill+BLHW | 11.49 | 0 | ~Year+UD.lev+Dist.WC+%UD.LF+%UD.BLHW |
| Landfill | 12.44 | 0 | ~Year+UD.lev+Dist.WC+%UD.LF |
| Landfill+BLHW+Weather | 14.69 | 0 | ~Year+UD.lev+Dist.WC+%UD.LF+%UD.BLHW+Temp+Press |
| Landfill*Sow | 18.04 | 0 | ~Year+UD.lev*Dist.WC*(%UD.LF+Age+Mass+Sounder) |
| Sow.int | 22.09 | 0 | ~Year+UD.lev*(Age+Mass+Sounder) |
| Sow | 22.71 | 0 | ~Year+UD.lev+Age+Mass+Sounder |
| Sow+BLHW | 23.71 | 0 | ~Year+UD.lev+Age+Mass+Sounder+%UD.BLHW |
| BLHW | 23.91 | 0 | ~Year+UD.lev+%UD.BLHW |
| BLHW.int | 24.03 | 0 | ~Year+UD.lev*%UD.BLHW |
| Sow+Weather | 25.71 | 0 | ~Year+UD.lev+Age+Mass+Sounder+Temp+Press |
| Weather | 26.12 | 0 | ~Year+UD.lev+Temp+Press |
| Sow+BLHW+Weather | 26.57 | 0 | ~Year+UD.lev+Age+Mass+Sounder+%UD.BLHW+Temp+Press |
| Null | 59.59 | 0 | ~1 |

^a^ UD.lev = utilization distribution (UD) contour level (i.e., 95% and 50% contours representing home ranges and core areas, respectively); Sounder = Sounder size; Mass = body mass (kg); Temp = temperature (C°); Press = barometric pressure (mb); HA.UD.LF = hectare (ha) of utilization distributions (UD) within the landfill boundary; %UD.LF = percent of UDs within the landfill; HA.UD.BLHW = ha of UDs within bottomland hardwood/wetland cover; %UD.BLHW = percent of UDs within bottomland hardwoods/wetland cover; and Dist.WC = distance from UD boundaries to the waste cells within the landfill (km).
